# Cholesterol dysregulation and microglial NLRP3 inflammasome activation after ischemic stroke: implications for neuroinflammation and functional recovery

**DOI:** 10.3389/fimmu.2026.1953192

**Published:** 2026-10-07

**Authors:** Jing Wang, Pingxu An, Chunyan Fu, Xingqi Su, Yan Sun, Di Ma, Xu Shi, Xinyu Hong

**Affiliations:** 1 Department of Cardiology and Cerebrovascular Diseases, Jilin Province Electric Power Hospital, Shuxun, Jilin, China; 2 Department of Oncological Neurosurgery, The First Hospital of Jilin University, Xinmin, Changchun, Jilin, China; 3 Lingyun Township Health Center, Dongliao, Liaoyuan, Jilin, China; 4 Department of Neurology and Neuroscience Center, The First Hospital of Jilin University, Xinmin, Changchun, Jilin, China

**Keywords:** cholesterol metabolism, immunometabolism, ischemic stroke, lipid droplets, microglia, neuroinflammation, NLRP3 inflammasome, oxysterols

## Abstract

Secondary neuroinflammation is an important determinant of tissue injury, repair, and neurological recovery after ischemic stroke, yet the lipid-metabolic environment in which this response develops remains incompletely integrated into current mechanistic models. This narrative review synthesizes PubMed-indexed, peer-reviewed literature published between 2010 and 2026 on post-stroke cholesterol metabolism, microglial lipid handling, NLRP3 inflammasome signaling, immunometabolic remodeling, therapeutic intervention, and recovery-related outcomes. Ischemic injury substantially remodels the local lipid environment through neuronal membrane disruption, myelin breakdown, oxidative lipid modification, altered cholesterol turnover, and blood–brain barrier dysfunction, generating a heterogeneous pool of free cholesterol, cholesteryl esters, lipid droplets, cholesterol crystals, oxysterols, and oxidized lipids. Stroke-specific studies increasingly support the emergence of cholesterol- and lipid-loaded microglial states and show that microglial lipid-processing capacity, including TREM2- and SREBP2-related mechanisms, can influence inflammatory and reparative responses. However, the causal connection between cholesterol dysregulation and NLRP3 activation remains only partly resolved. Direct stroke evidence supports the coexistence of microglial cholesterol overload and NLRP3-associated inflammatory changes, whereas detailed mechanisms involving cholesterol crystal-induced lysosomal injury, metabolic remodeling, and intracellular lipid stress remain partly dependent on cross-model evidence. Oxysterol effects are context dependent, and recent cholesterol-mobilization experiments identify mitochondrial DNA leakage–STING signaling as an alternative, more directly validated downstream pathway. Post-stroke microglia are also functionally heterogeneous, with distinct populations contributing to debris clearance, white matter repair, and neurological recovery. Therapeutically, systemic lipid management should be distinguished from experimental strategies that modify brain cholesterol handling or suppress inflammasome-associated inflammation, and no integrated cholesterol–microglia–NLRP3 treatment strategy has yet been clinically validated. Collectively, current evidence supports a proposed, evidence-stratified framework in which cholesterol-related lipid stress, microglial state, inflammasome susceptibility, and tissue repair partially converge rather than forming a single validated linear causal axis.

## 1 Introduction

Ischemic stroke remains a major cause of death and long-term disability worldwide. Recanalization therapies, including intravenous thrombolysis and mechanical thrombectomy, have substantially improved acute treatment for selected patients, yet many survivors continue to experience persistent motor, cognitive, and functional impairment. This gap between vascular recanalization and durable neurological recovery has increasingly shifted attention beyond acute tissue salvage toward the biological processes that govern inflammation resolution, tissue remodeling, and long-term repair (1, 2).

Neuroinflammation is a central component of this evolving response. Following ischemia–reperfusion injury, damaged cells release damage-associated molecular patterns, the blood–brain barrier becomes disrupted, resident microglia change their activation states, and peripheral immune populations can enter the injured tissue (1, 3, 4). The resulting inflammatory environment is temporally and spatially heterogeneous and includes signals arising from cellular injury, oxidative stress, ionic imbalance, mitochondrial dysfunction, and metabolic disturbance. The NLRP3 inflammasome is particularly relevant within this environment because canonical NLRP3 activation can integrate inflammatory priming with cellular stress signals such as K^+^ efflux, mitochondrial injury, reactive oxygen species, and lysosomal dysfunction (5, 6). Once assembled, the NLRP3–ASC–caspase-1 complex promotes IL-1β and IL-18 maturation and gasdermin D-mediated pyroptotic signaling, thereby contributing to inflammatory amplification after ischemic stroke (5–7). However, these mechanisms are not specific to cholesterol metabolism, and their contribution varies with disease stage, cellular source, and local tissue context.

Lipid metabolic remodeling adds an important but incompletely integrated dimension to this inflammatory environment. In the adult brain, cholesterol homeostasis is maintained predominantly through local synthesis and recycling, ApoE-containing lipoprotein transport, LXR-regulated ABCA1/ABCG1-mediated efflux, and CYP46A1-dependent conversion of cholesterol to 24S-hydroxycholesterol (8, 9). Ischemic injury can disturb this balance through neuronal membrane disruption, myelin breakdown, oxidative lipid modification, and altered cellular lipid processing. Blood–brain barrier dysfunction further modifies the metabolic and inflammatory environment but should not be interpreted as evidence for unrestricted transfer of circulating cholesterol into the brain parenchyma. Available studies support the emergence of a heterogeneous post-ischemic lipid environment containing free cholesterol, cholesteryl esters, lipid droplets, cholesterol crystals, oxysterols, and oxidized lipids, although the strength and biological interpretation of the supporting evidence differ among individual lipid species (10–13).

Microglia provide an important cellular context in which post-ischemic lipid remodeling and inflammatory signaling may intersect. As resident phagocytes, microglia remove dying cells, myelin debris, and lipid-rich material and process cholesterol through lysosomal degradation, esterification, lipid-droplet storage, intracellular trafficking, and cholesterol efflux (8, 10, 11). When persistent lipid exposure exceeds intracellular processing capacity, free cholesterol, cholesteryl esters, and lipid droplets may accumulate and contribute to lipid-loaded and metabolically stressed microglial states (10, 11). These responses should not, however, be regarded as a single fixed or uniformly detrimental phenotype. Microglial functions change over time and can contribute both to inflammatory responses and to debris clearance, remodeling, and neurological repair (2). Cholesterol-derived signals should likewise not be considered uniformly pro-inflammatory, because their effects can depend on molecular species, cellular context, and disease stage (12, 13).

How these lipid-associated microglial states intersect with NLRP3 signaling remains an important unresolved question. Current evidence supports the biological plausibility that cholesterol-related lipid stress can modify microglial inflammatory susceptibility, but it does not establish a single obligatory sequence from cholesterol accumulation to NLRP3 activation and failed neurological recovery. Detailed mechanisms such as cholesterol crystal-associated lysosomal injury remain supported partly by evidence from non-stroke inflammatory systems, whereas cholesterol-derived metabolites can also exert context-dependent or even protective effects (12, 13). This review therefore evaluates the cholesterol–microglia–NLRP3 relationship as a proposed, evidence-stratified and stage-dependent framework rather than as a validated linear causal axis. We first examine the sources and temporal evolution of post-ischemic cholesterol dysregulation, then consider microglial lipid-processing and immunometabolic states and their potential convergence with NLRP3-associated signaling. Finally, we assess how these partially overlapping processes may influence tissue repair and functional recovery and discuss therapeutic strategies according to their stage of intervention and level of supporting evidence.

### 1.1 Literature identification and scope of evidence

This article is a narrative review informed by targeted searches of PubMed, which was the only database used for literature identification. English-language, peer-reviewed articles published between January 2010 and August 2026 were considered. Search terms were centered on ischemic stroke and cerebral ischemia in combination with topics relevant to the present review, including cholesterol metabolism, oxysterols, microglia, TREM2, lipid droplets, NLRP3 inflammasome, pyroptosis, neuroinflammation, and functional recovery. The literature search was updated during manuscript revision to identify recently published studies relevant to post-stroke cholesterol metabolism, microglial lipid handling, and inflammasome-related inflammation. Preprints, conference abstracts, unpublished studies, and other non-peer-reviewed materials were not considered.

The narrative synthesis focused primarily on peer-reviewed original experimental, translational, and clinical studies relevant to ischemic stroke. Particular attention was given to studies addressing post-ischemic cholesterol and lipid remodeling, microglial lipid handling and metabolic states, inflammasome activation, and recovery-related outcomes. Peer-reviewed review articles were also consulted to provide background context, integrate established concepts, and identify relevant primary studies; when available, primary studies were preferentially used to support specific mechanistic claims.

Because direct stroke-specific evidence was not available for every mechanistic link discussed in this review, selected studies from related fields, including macrophage biology, atherosclerosis, and other neuroinflammatory conditions, were considered when they provided mechanistic information directly relevant to the proposed framework. Such findings were treated as indirect or cross-model evidence rather than as equivalent to stroke-specific causal evidence. Accordingly, the literature identification process was intended to support a targeted narrative synthesis rather than a systematic or scoping review, and no PRISMA-based study-selection procedure or formal risk-of-bias assessment was performed.

## 2 Brain cholesterol homeostasis and post-ischemic dysregulation

Brain cholesterol homeostasis is maintained through coordinated local synthesis, intercellular transport, intracellular storage, efflux, and oxysterol-mediated clearance. Ischemic injury perturbs several of these processes simultaneously through neuronal membrane disruption, myelin breakdown, oxidative lipid modification, altered cholesterol turnover, and blood–brain barrier dysfunction. The resulting lipid environment is heterogeneous and may include free cholesterol, cholesteryl esters, lipid droplets, cholesterol crystals, oxysterols, and other oxidized lipid species. Importantly, the evidence supporting the formation and inflammatory relevance of these lipid species is not uniform. Some changes have been demonstrated directly in experimental stroke, whereas several proposed downstream inflammatory mechanisms are supported mainly by studies in other myeloid or lipid-rich inflammatory systems. This section therefore distinguishes post-stroke lipid remodeling from mechanistic extrapolations regarding its downstream inflammatory consequences.

### 2.1 Cholesterol homeostasis in the normal brain

Brain cholesterol metabolism is characterized by both abundance and compartmentalization. Cholesterol is highly enriched in myelin and neuronal membranes, where it contributes to membrane organization, myelin stability, synaptic function, vesicular trafficking, and neuronal signaling (8). Because the adult central nervous system contains a large, locally maintained cholesterol pool, disruption of neuronal membranes, myelin integrity, or intracellular lipid trafficking can substantially alter the lipid burden encountered by surrounding glial cells.

Unlike most peripheral tissues, the adult brain relies predominantly on local cholesterol synthesis and recycling because the blood–brain barrier restricts the direct exchange of circulating cholesterol-rich lipoproteins with the brain parenchyma (8). Astrocyte-derived ApoE-containing lipoprotein particles contribute to intercellular cholesterol transport and can be taken up by neurons and glial cells through receptors including LDLR and LRP1 (8). This local transport system supports membrane renewal, synaptic remodeling, and myelin maintenance while limiting the extent to which brain cholesterol homeostasis is determined directly by circulating cholesterol concentrations.

Excess brain cholesterol is removed principally through enzymatic conversion and cellular efflux pathways. CYP46A1 converts cholesterol to 24S-hydroxycholesterol (24S-HC), which can cross the blood–brain barrier and enter the peripheral circulation (8, 9), whereas LXR-regulated transporters, including ABCA1 and ABCG1, promote cellular cholesterol efflux and ApoE-dependent lipoprotein remodeling (8). Together, these pathways maintain local cholesterol balance under physiological conditions. Their disruption after ischemic injury is therefore relevant not only to total cholesterol abundance but also to the cellular distribution, chemical form, and clearance of cholesterol-derived lipids.

### 2.2 Sources and accumulation of cholesterol-related lipids after ischemic stroke

Ischemic injury produces substantial remodeling of the local lipid environment through cellular membrane disruption, myelin injury, and altered lipid processing. Experimental lipidomic studies have demonstrated that these changes are spatially and temporally dynamic rather than representing a uniform increase in total lipid content. In mouse models of cerebral ischemia, cholesteryl esters and other neutral lipid species change across post-ischemic brain regions and time points, accompanied by increased lipid droplet accumulation and PLIN2 expression in microglia (11). Lipid-droplet-rich microglia in these models also exhibit increased expression of inflammatory mediators, supporting a direct association between post-ischemic lipid remodeling and altered microglial inflammatory states (11).

More direct evidence for microglial cholesterol accumulation has emerged from studies of the chronic post-stroke phase. Zhao et al. identified persistent cholesterol accumulation and extensive cholesterol-metabolic reprogramming in microglia after experimental ischemic stroke, including a distinct population of stroke-associated foamy microglia (10). Importantly, intracerebral administration of either free cholesterol or cholesterol crystals reproduced sustained microglial activation, whereas genetic or pharmacological enhancement of CYP46A1-mediated cholesterol clearance reduced cholesterol overload and chronic neuroinflammation and promoted white matter repair and functional recovery (10). These findings provide stroke-specific experimental support for a functional relationship between excessive cholesterol burden and persistent microglial inflammatory activation, particularly during the chronic phase.

Recent evidence further strengthens the existence of cholesterol-loaded microglial states after ischemic stroke. In a 2026 study using dMCAO, Cheng et al. observed progressive accumulation of free cholesterol in peri-infarct microglia, together with cholesterol crystal deposition and neutral lipid droplet formation (14). Cholesterol crystal-containing microglia showed substantial overlap with lipid-droplet-rich cells, and single-cell analysis identified an IFITM3+ microglial population characterized by increased ACAT1 expression, cholesterol esterification, cholesterol crystals, and lipid droplet accumulation (14). Pharmacological cholesterol mobilization with 2-hydroxypropyl-β-cyclodextrin reduced microglial free cholesterol, ACAT1 expression, and lipid droplet accumulation and was accompanied by attenuation of inflammatory signaling and improved neurological outcomes (14).

Together, these studies provide increasingly direct stroke-specific evidence that ischemic injury is followed by dynamic lipid remodeling and the emergence of cholesterol- and lipid-droplet-loaded microglial states (10, 11, 14). However, the evidence is strongest for the existence and inflammatory relevance of post-stroke microglial lipid overload; it does not establish that all downstream inflammatory effects are mediated specifically through the NLRP3 inflammasome. The mechanisms by which microglia process this lipid burden, and how failure of these processes reshapes inflammatory signaling, are considered in Section 3.

### 2.3 Cholesterol crystals and oxysterols: evidence for inflammatory signaling after stroke

Cholesterol accumulation after ischemic injury can generate chemically and structurally distinct lipid species with different inflammatory properties. Among these, cholesterol crystals and oxysterols are particularly relevant because both have been linked to innate immune signaling, although the strength and disease context of the supporting evidence differ substantially. Recent stroke studies have strengthened evidence for the presence of cholesterol crystals in lipid-loaded microglia, whereas several downstream mechanisms connecting cholesterol-derived lipids to inflammasome activation remain informed largely by non-stroke inflammatory models.

#### 2.3.1 Cholesterol crystals

For this reason, cholesterol crystals should be interpreted as plausible lipid-derived particulate danger signals after stroke, but not as an isolated explanation for NLRP3 activation. Their contribution probably depends on lesion stage, local cholesterol burden, myeloid cell phenotype, and the efficiency of cholesterol clearance. In the post-ischemic brain, cholesterol crystals are more likely to cooperate with damage-associated molecular patterns, myelin debris, oxidized lipids, lysosomal stress, and mitochondrial injury than to act through a single independent pathway.

Cholesterol crystals are well-established particulate activators of the NLRP3 inflammasome in macrophage and atherosclerosis models. In these systems, crystal uptake can induce lysosomal membrane damage and release of lysosomal contents, thereby promoting NLRP3 inflammasome activation and downstream IL-1β production (13). This mechanism provides a biologically plausible route through which excessive intracellular cholesterol could contribute to inflammatory signaling, but it should not be considered equivalent to direct demonstration of the same pathway in post-stroke microglia.

Stroke-specific evidence for cholesterol crystal accumulation has nevertheless strengthened substantially. Chronic experimental stroke has been associated with persistent cholesterol accumulation and cholesterol-loaded, foam-like microglial states, and experimental manipulation of cholesterol burden influences chronic neuroinflammation and recovery (10). More recently, Cheng et al. directly demonstrated progressive cholesterol crystal deposition within Iba1+ microglia after dMCAO, together with free cholesterol and lipid droplet accumulation (14). Cholesterol crystal-containing microglia were frequently lipid-droplet rich and showed increased NLRP3 expression, whereas pharmacological cholesterol mobilization reduced lipid burden and the abundance of NLRP3-positive microglia (14).

These findings provide direct stroke-specific evidence that cholesterol crystals can accumulate within microglia and are associated with an inflammasome-related inflammatory state. However, they do not yet establish the complete sequence of cholesterol crystal uptake → lysosomal rupture → NLRP3 assembly → caspase-1 activation specifically in post-ischemic microglia. At present, the lysosomal mechanism is therefore best regarded as a cross-model mechanistic explanation that is compatible with, but not fully demonstrated by, the available stroke evidence (13, 14).

#### 2.3.2 Oxysterols

Oxysterols are generated through enzymatic cholesterol oxidation or non-enzymatic oxidation under conditions of oxidative stress. Their biological effects are heterogeneous and depend on the specific oxysterol, concentration, cell type, and disease stage. This distinction is particularly important in ischemic stroke because available evidence does not support a uniformly pro-inflammatory role for cholesterol oxidation products.

25-Hydroxycholesterol (25-HC) illustrates this context dependence. In an experimental cerebral ischemia–reperfusion model, 25-HC reduced ischemic injury through inhibition of STING signaling, providing direct stroke-specific evidence for an acute protective effect (12). By contrast, evidence from a non-stroke neurological model has linked microglial CH25H/25-HC signaling to altered lipid metabolism and NLRP3 inflammasome activation (15). The latter finding supports the biological possibility that CH25H/25-HC signaling can interact with inflammasome pathways, but it should not be extrapolated as evidence that 25-HC activates microglial NLRP3 after ischemic stroke.

A similar limitation applies to 7-ketocholesterol (7-KC). Studies in retinal microglia and other oxidized-lipid inflammatory systems have shown that 7-KC can promote microglial activation and TLR4-associated inflammatory signaling (16, 17). However, direct evidence demonstrating a causal role for 7-KC in microglial NLRP3 activation after ischemic stroke remains lacking. These studies should therefore be viewed as cross-model mechanistic evidence rather than stroke-specific validation.

Taken together, cholesterol crystals and oxysterols illustrate why cholesterol-derived inflammatory signaling after stroke should not be represented as a single linear pathway. Evidence for post-stroke microglial cholesterol crystal accumulation is increasingly direct, whereas the downstream lysosomal–NLRP3 mechanism remains partly extrapolated from macrophage and atherosclerosis models. Oxysterol effects are even more context dependent, with direct stroke evidence supporting protective as well as potentially inflammatory actions. These distinctions argue for viewing cholesterol-derived lipids as heterogeneous modulators of post-ischemic inflammatory susceptibility rather than as uniformly acting NLRP3 agonists.

### 2.4 Blood–brain barrier disruption, peripheral lipid background, and myeloid cell infiltration

Blood–brain barrier (BBB) disruption is a major component of ischemia–reperfusion injury and increases the exposure of injured brain tissue to blood-derived proteins, inflammatory mediators, and infiltrating immune cells (1, 4). However, BBB dysfunction should not be interpreted as evidence for unrestricted transfer of circulating cholesterol into the brain parenchyma. The adult brain retains a largely compartmentalized cholesterol pool, and the lipid burden encountered by microglia after stroke is also generated locally through membrane disruption, myelin breakdown, lipid oxidation, and impaired cellular lipid processing. BBB disruption is therefore better viewed as a modifier of the post-ischemic metabolic and inflammatory environment rather than as the primary source of brain cholesterol accumulation.

Clinical observations nevertheless indicate that systemic lipid handling is associated with stroke severity and outcome. In patients with acute ischemic stroke, reduced HDL cholesterol efflux capacity and altered HDL phospholipid composition were associated with greater neurological severity and functional dependency at discharge (18). These findings suggest that functional measures of peripheral cholesterol handling may provide prognostic information, but they do not demonstrate that systemic cholesterol efflux directly regulates cholesterol accumulation or inflammasome activation in post-stroke microglia. Similarly, lipidomic analysis of paired thrombi and plasma from patients undergoing endovascular thrombectomy identified stroke-etiology-associated lipid signatures in thrombi, whereas significant differences were not detected in plasma (19). This distinction further cautions against using circulating lipid profiles as direct surrogates for the lipid environment within the ischemic brain.

BBB disruption also permits recruitment of peripheral myeloid cells, adding another layer of complexity to the cellular response after stroke (4, 20). Resident microglia and infiltrating monocyte-derived macrophages can share functions related to debris clearance, lipid metabolism, and inflammatory signaling, but their contributions are not interchangeable and may vary with disease stage. Importantly, infiltrating macrophages are not uniformly pro-inflammatory. Recent experimental evidence identified a population of repair-associated monocyte-derived macrophages with enhanced lipid-metabolic and pro-angiogenic programs after ischemic stroke; expansion or transplantation of these cells was associated with improved white matter and vascular repair and better functional recovery (20). Thus, recruitment of peripheral myeloid cells should not be represented simply as an amplifier of lipid-driven inflammation.

Taken together, peripheral lipid status, BBB dysfunction, and infiltrating immune cells can modify the post-ischemic metabolic environment, but the available evidence does not establish a direct peripheral cholesterol–microglial NLRP3 pathway. These factors are therefore best considered contextual modifiers of the proposed cholesterol–microglia inflammatory framework rather than equivalent components of a linear causal chain.

### 2.5 Evidence synthesis and unresolved links

Collectively, current stroke-specific evidence supports substantial remodeling of cholesterol-related lipids after ischemic injury. Experimental studies have demonstrated dynamic changes in brain lipid profiles, lipid droplet accumulation in microglia, persistent microglial cholesterol overload, and the emergence of cholesterol-loaded or foam-like microglial states (10, 11, 14). More recent work has also directly identified free cholesterol and cholesterol crystals within peri-infarct microglia and shown that interventions that reduce cholesterol burden can attenuate inflammatory responses and improve neurological recovery (10, 14). These findings establish post-ischemic microglial cholesterol dysregulation as a biologically relevant component of the injured brain environment rather than merely a passive consequence of tissue damage.

However, the downstream causal links remain less completely resolved. In particular, the full sequence linking cholesterol accumulation or crystallization to lysosomal injury, NLRP3 inflammasome assembly, and recovery failure has not yet been demonstrated within a single stroke-specific experimental framework. Key elements of this pathway remain supported by cross-model mechanistic evidence (13), while oxysterol effects can be directionally heterogeneous, as illustrated by the protective action of 25-hydroxycholesterol through STING inhibition in cerebral ischemia–reperfusion (12). The next section therefore focuses on how microglia process the post-ischemic lipid burden and how altered lipid-handling states may shape inflammatory susceptibility. The sources and temporal evolution of post-stroke cholesterol dysregulation are summarized in **Figure 1**.

**Figure 1:**
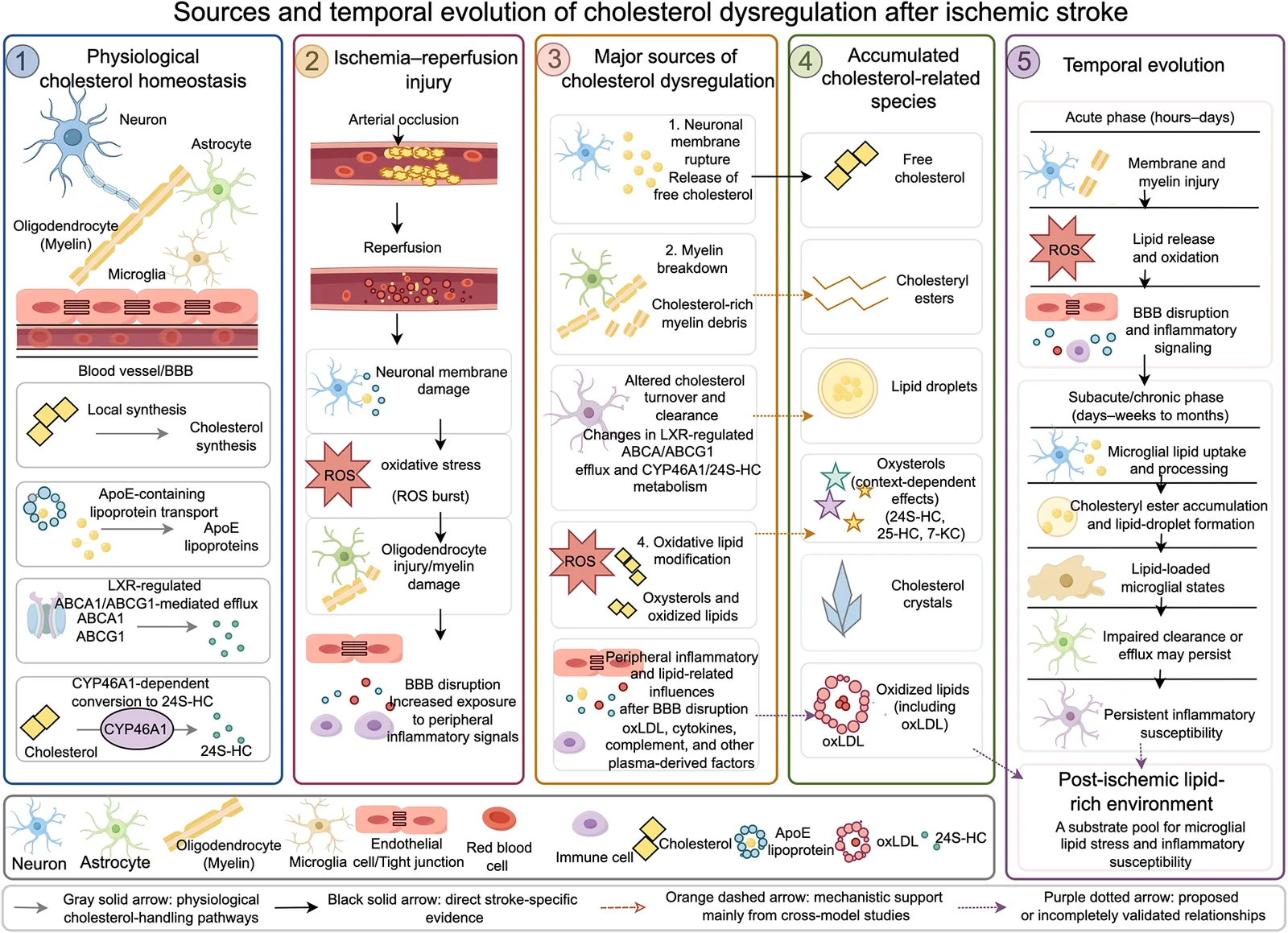
Sources and temporal evolution of cholesterol dysregulation after ischemic stroke. Under physiological conditions, brain cholesterol homeostasis is maintained through local synthesis and recycling, ApoE-containing lipoprotein transport, LXR-regulated ABCA1/ABCG1-mediated efflux, and CYP46A1-dependent conversion of cholesterol to 24S-hydroxycholesterol (24S-HC). Ischemia–reperfusion injury disrupts this balance through neuronal membrane damage, myelin breakdown, oxidative lipid modification, altered cholesterol turnover, and blood–brain barrier (BBB) dysfunction. These processes generate a heterogeneous post-ischemic lipid environment containing free cholesterol, cholesteryl esters, lipid droplets, cholesterol crystals, oxysterols, and oxidized lipids. During the acute phase, membrane and myelin injury, lipid release and oxidation, and BBB disruption predominate, whereas subacute and chronic stages increasingly involve microglial lipid uptake and processing, intracellular lipid accumulation, lipid-loaded microglial states, and potentially persistent inflammatory susceptibility. Lipid uptake and storage may initially support adaptive debris clearance, whereas persistent lipid burden or insufficient processing capacity may contribute to maladaptive lipid stress. BBB dysfunction and peripheral factors are represented as contextual modifiers rather than as evidence for unrestricted entry of circulating cholesterol into the brain. Gray solid arrows indicate physiological cholesterol-handling pathways; black solid arrows indicate relationships directly supported by ischemic-stroke evidence; orange dashed arrows indicate mechanistic relationships supported mainly by cross-model studies; and purple dotted arrows indicate proposed or incompletely validated relationships. ApoE, apolipoprotein E; LXR, liver X receptor; ABCA1/ABCG1, ATP-binding cassette transporters A1/G1; CYP46A1, cholesterol 24-hydroxylase; 24S-HC, 24S-hydroxycholesterol; BBB, blood–brain barrier; oxLDL, oxidized low-density lipoprotein. By Figdraw.

## 3 Microglial lipid handling and immunometabolic remodeling after ischemic stroke

After ischemic stroke, microglia are exposed to a lipid environment that differs substantially from physiological tissue turnover. Damaged neuronal membranes, cholesterol-rich myelin debris, oxidized lipids, and other cellular remnants increase the amount and diversity of lipid substrates that must be processed by resident phagocytes (10, 11, 14). Uptake of this material is initially an adaptive component of debris clearance and tissue remodeling. However, when lipid influx persists or exceeds intracellular processing and efflux capacity, microglia can accumulate free cholesterol, cholesteryl esters, and lipid droplets and adopt lipid-loaded states associated with altered inflammatory and reparative functions (10, 11, 14).

Importantly, lipid-loaded microglia should not be treated as a single fixed or uniformly pro-inflammatory phenotype. Stroke-specific transcriptomic and metabolic studies instead indicate heterogeneous and temporally evolving responses involving lipid uptake, esterification, storage, hydrolysis, and efflux (10, 11). This section therefore focuses on the cellular processes that determine whether post-ischemic lipid uptake remains adaptive or progresses toward metabolic stress. Particular attention is given to cholesterol-processing pathways, TREM2–ApoE-related lipid handling, lipid droplet regulation, autophagy-associated mechanisms, and broader immunometabolic remodeling. Their potential convergence on NLRP3 inflammasome activation is considered separately in Section 4.

### 3.1 Microglial lipid uptake and cholesterol-processing capacity

Microglia contribute to the removal of dying cells, membrane fragments, and myelin-derived lipids after ischemic injury. Once internalized, cholesterol-containing material must be coordinated across lysosomal degradation, intracellular cholesterol trafficking, esterification and lipid droplet storage, and cholesterol efflux pathways (8, 10, 11). These processes are not simply mechanisms of lipid disposal. Rather, they determine whether excess free cholesterol is safely redistributed or stored, or instead accumulates to levels that impose cellular stress.

Direct stroke-specific evidence indicates that this cholesterol-processing network is dynamically remodeled after ischemic injury. Zhao et al. demonstrated increases in both free cholesterol and cholesteryl ester metabolism in post-stroke microglia, together with temporal regulation of genes involved in cholesterol uptake, esterification, hydrolysis, and efflux, including Ldlr, Soat1, Nceh1, Abca1, Abcg1, and Apoe (10). Single-cell analysis further identified stroke-associated foamy microglial populations enriched in lipid-processing genes such as Apoe, Lpl, Trem2, Npc2, Nceh1, and Fabp5 (10). These findings indicate that post-stroke cholesterol accumulation is accompanied by active metabolic adaptation rather than simple passive lipid deposition.

Recent data provide additional evidence that this adaptive capacity can become insufficient. Cheng et al. identified an IFITM3+ microglial state after dMCAO characterized by increased ACAT1 expression, cholesterol esterification, free cholesterol accumulation, cholesterol crystals, and lipid droplets (14). Pharmacological mobilization of cholesterol reduced microglial free cholesterol, ACAT1 expression, and lipid droplet burden, supporting a functional relationship between intracellular cholesterol handling and the maintenance of lipid-loaded microglial states (14). Together with earlier lipidomic studies showing dynamic post-ischemic cholesteryl ester and lipid droplet remodeling (11), these observations provide direct evidence that the balance among cholesterol uptake, storage, turnover, and efflux is altered after stroke.

Studies in macrophage and other sterile inflammatory systems identify CD36 as a candidate mediator of modified-lipid uptake and intracellular inflammatory stress (21). CD36-dependent uptake can facilitate the conversion of soluble modified lipids into particulate intracellular material and promote lysosomal inflammatory stress (21). However, the contribution of CD36 to lipid uptake in post-stroke microglia remains incompletely defined. It should therefore be regarded as a candidate uptake mechanism rather than direct evidence for a stroke-specific CD36–cholesterol inflammatory pathway.

Overall, current evidence supports a model in which microglial responses to post-ischemic lipid exposure are determined not simply by the amount of lipid taken up, but by the capacity to traffic, esterify, store, hydrolyze, and export cholesterol-containing material. Failure of this processing capacity provides a plausible transition from adaptive debris clearance to persistent lipid stress. The regulatory role of TREM2 in this balance is considered next.

### 3.2 TREM2-related lipid handling and lipid droplet regulation

TREM2 is an important regulator of microglial adaptation to lipid-rich and phagocytic challenges. In the post-ischemic brain, its relevance appears to extend beyond inflammatory signaling alone, because TREM2 influences debris phagocytosis, cholesterol turnover, and lipid droplet homeostasis. Stroke-specific studies therefore support viewing TREM2 primarily as a regulator of microglial lipid-processing capacity rather than as a simple marker of an anti-inflammatory state (22, 23).

Direct evidence for this role comes from experimental ischemic models in which TREM2 deficiency impaired microglial phagocytosis and promoted cholesteryl ester accumulation and PLIN2-positive lipid droplet formation (22). TREM2 knockdown increased expression of lipid-storage-associated genes, including Plin2 and Soat1, while reducing genes involved in cholesterol hydrolysis, intracellular trafficking, and efflux, including Lipa, Apoe, Abca1, Nceh1, and Npc2 (22). These metabolic changes were accompanied by a shift toward a more inflammatory microglial state and loss of the neuroprotective effect of microglia in neuron–microglia co-culture experiments (22). Together, these findings provide direct stroke-related evidence that inadequate TREM2 activity can impair cholesterol processing and favor the persistence of lipid-droplet-rich microglia.

TREM2 also participates in phagocytic responses to lipid-associated ligands after cerebral ischemia. Sphingosine-1-phosphate (S1P) has been identified experimentally as a TREM2 ligand, and S1P–TREM2 signaling enhanced microglial phagocytosis and reduced ischemic brain injury in a TREM2-dependent manner (23). In the same study, microglia-derived ApoE carrying S1P contributed to recognition and clearance of apoptotic neurons, providing stroke-specific evidence that TREM2 activity can interact functionally with ApoE-associated lipid transport during phagocytic clearance (23). This finding supports an interaction between TREM2-dependent sensing and ApoE-related lipid handling, but does not establish a universal linear TREM2–ApoE signaling cascade after stroke.

Lipid droplet formation itself should also be interpreted in a context-dependent manner. Lipid droplets can provide a temporary buffering mechanism by sequestering excess neutral lipids and limiting exposure to potentially toxic free lipid species. However, sustained lipid accumulation or impaired mobilization may indicate failure of intracellular lipid turnover. In ischemic models, lipid droplet accumulation is associated with altered microglial inflammatory responses (11, 22), while chronic stroke studies identify lipid-loaded and foam-like microglial populations in which Trem2, Apoe, Lpl, Nceh1, and related lipid-processing genes are co-expressed with inflammatory programs (10). These observations suggest that lipid droplets are better viewed as markers and regulators of an evolving metabolic state rather than as intrinsically protective or pathogenic structures.

Overall, the available stroke-specific evidence supports TREM2 as an important determinant of whether microglia can accommodate the lipid burden generated by ischemic tissue injury. Reduced TREM2 function can impair phagocytosis, cholesterol hydrolysis and efflux, and lipid droplet turnover, whereas preserved TREM2-related activity may facilitate debris clearance and metabolic adaptation (22, 23). Nevertheless, the extent to which TREM2-mediated lipid handling directly controls NLRP3 inflammasome activation remains insufficiently established in stroke. TREM2 should therefore be positioned upstream as a regulator of lipid-processing state, with its potential influence on inflammasome susceptibility considered separately from direct NLRP3 signaling.

### 3.3 SREBP2, autophagy, and intracellular lipid processing

In addition to lipid uptake and efflux, the intracellular cholesterol burden of microglia is influenced by transcriptional programs that regulate lipid synthesis and organelle turnover. Sterol regulatory element-binding protein 2 (SREBP2) is a major regulator of cholesterol biosynthesis and has recently been implicated in post-stroke microglial lipid remodeling. In experimental ischemic stroke, SREBP2 expression was markedly increased after cerebral ischemia and was associated with cholesterol-related lipid accumulation, inflammatory activation, and changes in autophagy-related signaling (24).

Recent work by Wei et al. provided direct stroke-specific evidence linking SREBP2 to this metabolic response. Using MCAO and complementary *in vitro* ischemic models, the authors reported that SREBP2 upregulation was accompanied by increased cholesterol and cholesteryl ester accumulation and altered expression of autophagy-related proteins, including LC3 and p62 (24). Manipulation of SREBP2, together with pharmacological modulation of autophagy, indicated that autophagy-related processes contributed to the effects of SREBP2 on cholesterol-associated lipid accumulation and inflammatory mediator production (24). Notably, SREBP2 suppression reduced the excessive autophagy-associated response as well as cholesterol-related lipid burden and inflammatory signaling, suggesting that autophagy in this setting cannot be interpreted simply as a uniformly protective lipid-clearance pathway.

The functional consequences extended beyond lipid measurements. In microglia–neuron co-culture experiments, interference with microglial SREBP2 improved the survival of OGD/R-injured neurons and reduced neuronal apoptosis, while *in vivo* SREBP2 intervention was associated with reduced ischemic injury and improved neurological recovery after MCAO (24). These findings support a functional relationship among SREBP2-dependent lipid dysregulation, autophagy-related responses, neuroinflammation, and tissue injury after stroke.

This SREBP2–autophagy interaction adds an intracellular regulatory layer to the cholesterol-processing mechanisms described above. Whereas TREM2-related pathways primarily influence lipid recognition, phagocytosis, turnover, and efflux (22), SREBP2 may additionally regulate the balance between endogenous cholesterol synthesis, intracellular lipid storage, and autophagy-associated processing. However, the available evidence does not establish that SREBP2-driven lipid remodeling activates the NLRP3 inflammasome directly. Its current relevance to the proposed framework is therefore strongest at the level of cholesterol dysregulation → microglial metabolic stress and inflammatory remodeling, with the potential connection to NLRP3 requiring separate validation.

### 3.4 Lipid-associated microglial state transitions and immunometabolic remodeling

Post-stroke microglial responses cannot be adequately represented by a binary pro-inflammatory versus repair-associated classification. Single-cell, single-nucleus, and spatial transcriptomic studies instead reveal multiple temporally and regionally distinct myeloid states after ischemic injury (25–27). Within this heterogeneous landscape, stroke-associated microglia can co-express lipid-handling genes, including Apoe, Trem2, Lpl, Fabp5, and Abca1, together with inflammatory, chemokine, and antigen-presentation programs (10, 25, 26). Chronic stroke studies further identify cholesterol-loaded and foam-like microglial populations with persistent lipid-metabolic remodeling (10). These findings suggest that altered lipid handling is embedded within broader microglial state transitions rather than defining a single discrete inflammatory phenotype.

Lipid accumulation also occurs within a wider reorganization of cellular energy metabolism. Ischemia imposes hypoxia, oxidative stress, mitochondrial dysfunction, and increased demands for phagocytosis and lipid processing, all of which can alter the balance between oxidative phosphorylation and glycolytic metabolism. Stroke-focused studies support an important role for mitochondrial metabolic capacity in determining microglial inflammatory behavior. For example, microglial PGC-1α, a regulator of mitochondrial biogenesis and metabolic homeostasis, is dynamically altered after ischemic stroke, and microglia-specific enhancement of PGC-1α suppresses neuroinflammation and reduces ischemic brain injury (28). These findings indicate that maintenance of mitochondrial and metabolic fitness can influence whether activated microglia sustain or resolve inflammatory responses, although this mechanism is not specific to cholesterol metabolism.

Conversely, sustained lipid burden may coexist with glycolytic and mitochondrial stress. Current stroke literature supports metabolic reprogramming as an important feature of ischemic inflammation, but direct evidence linking cholesterol accumulation itself to a defined glycolytic program in post-stroke microglia remains incomplete (29). Mechanisms involving HIF-1α-dependent glycolysis, tricarboxylic acid cycle remodeling, succinate accumulation, PKM2 signaling, and mitochondrial reactive oxygen species have been implicated in inflammatory myeloid metabolism, but much of the detailed causal evidence comes from non-stroke or non-microglial systems (30–32). These pathways therefore provide mechanistic hypotheses for how lipid stress and altered bioenergetics might interact after stroke rather than establishing a cholesterol-specific metabolic sequence.

Taken together, the available evidence supports a dynamic model in which post-ischemic microglial states are shaped jointly by lipid-processing capacity, mitochondrial fitness, and broader metabolic demands. TREM2-related lipid handling, SREBP2-associated intracellular cholesterol regulation, lipid droplet turnover, and mitochondrial metabolic adaptation may determine whether microglia accommodate the lipid burden or develop persistent metabolic stress (10, 22, 24, 28). Importantly, these metabolic changes should not be equated directly with NLRP3 activation. The mechanisms by which glycolytic remodeling, mitochondrial stress, lysosomal dysfunction, and cholesterol-derived signals may converge on inflammasome priming and assembly are addressed in Section 4.

### 3.5 Evidence synthesis and unresolved links

Current stroke-specific evidence supports the view that microglial lipid accumulation after ischemic injury is an active and dynamically regulated process rather than a passive consequence of tissue damage. Post-stroke microglia undergo changes in cholesterol uptake, esterification, intracellular storage, hydrolysis, and efflux, while TREM2-dependent lipid handling, lipid droplet regulation, SREBP2-associated cholesterol metabolism, and mitochondrial metabolic fitness can influence their capacity to accommodate the increased lipid burden (10, 11, 14, 22, 24, 28). These responses appear to be context dependent: lipid uptake and storage can initially support debris clearance and metabolic adaptation, whereas persistent lipid accumulation or insufficient processing capacity can favor cholesterol-loaded, foam-like, or otherwise metabolically stressed microglial states (10, 11, 22).

However, the causal transition from impaired lipid handling to inflammasome activation remains incompletely defined in ischemic stroke. Direct stroke studies establish microglial cholesterol accumulation, lipid droplet remodeling, altered cholesterol-processing programs, and inflammatory state changes, but they do not yet demonstrate that these events form a single obligatory sequence leading to NLRP3 activation. Detailed mechanisms involving cholesterol crystal-induced lysosomal injury, PKM2-dependent glycolytic signaling, organelle cholesterol trafficking, and related inflammasome-regulatory pathways are supported to varying degrees by macrophage, atherosclerosis, or other inflammatory models (13, 31, 33). These mechanisms therefore provide plausible routes of convergence rather than fully validated stroke-specific steps. Section 4 examines this distinction directly by separating established mechanisms of NLRP3 activation in ischemic stroke from cholesterol-related mechanisms that remain partly supported by cross-model evidence.

## 4 NLRP3 inflammasome activation in ischemic stroke

### 4.1 Canonical NLRP3 inflammasome priming and activation after ischemic stroke

The NLRP3 inflammasome is a cytosolic multiprotein complex composed principally of the NLRP3 sensor, the adaptor ASC, and pro-caspase-1. Canonical NLRP3 activation is commonly conceptualized as a two-step process involving priming and activation (5, 6). Priming is typically initiated by inflammatory receptors, including Toll-like receptors, tumor necrosis factor receptors, and interleukin-1 receptors, and promotes NF-κB-dependent expression of NLRP3 and pro-IL-1β. A subsequent activation step is triggered by cellular stress signals such as K^+^ efflux, extracellular ATP/P2X7 signaling, mitochondrial dysfunction, mitochondrial reactive oxygen species, lysosomal membrane injury, and disturbed ionic homeostasis, leading to NLRP3 oligomerization, ASC recruitment, and caspase-1 activation (5, 6).

Ischemia–reperfusion injury can provide both types of signals within the same evolving lesion environment. Damage-associated molecular patterns released from injured or dying cells, including ATP, HMGB1, and nucleic acids, can contribute to inflammatory priming, whereas ionic imbalance, mitochondrial injury, oxidative stress, and lysosomal dysfunction provide potential activation signals (5, 6). These processes may overlap temporally rather than occurring as completely discrete events after stroke. Importantly, none of these mechanisms is specific to cholesterol metabolism. Cholesterol-derived signals should therefore be considered as one subset of metabolic and organelle stressors that may interact with the broader NLRP3-activating environment, rather than as an independent two-signal pathway. Their stroke-specific and cross-model evidence is evaluated in the following section.

### 4.2 Convergence of cholesterol-related and cellular stress signals on NLRP3

Cholesterol-related signals are best viewed as components of a broader stress environment that can modify NLRP3 responsiveness after ischemic stroke rather than as a separate cholesterol-specific activation pathway. The strongest direct stroke-specific evidence currently links microglial cholesterol accumulation with an NLRP3-associated inflammatory state. In experimental stroke, cholesterol-loaded microglia containing free cholesterol, cholesterol crystals, and lipid droplets show increased NLRP3 expression, and pharmacological mobilization of cholesterol reduces both the microglial lipid burden and the abundance of NLRP3-positive microglia (14). Importantly, however, these findings do not establish NLRP3 as the necessary downstream mediator of cholesterol-induced injury. In the same study, the most extensively validated downstream mechanism involved mitochondrial DNA leakage and STING signaling, supported by pharmacological STING inhibition and microglia-targeted STING knockdown (14). Thus, current stroke evidence supports an association between cholesterol dysregulation and an NLRP3-related inflammatory phenotype, while leaving the specific causal contribution of NLRP3 incompletely resolved.

Cholesterol crystals provide the most established mechanistic link between cholesterol overload and NLRP3 activation, although much of the detailed causal evidence originates outside stroke. In macrophage and atherosclerosis models, phagocytosed cholesterol crystals can disrupt lysosomal integrity, promote release of lysosomal contents, and activate the NLRP3 inflammasome (13). The recent demonstration of cholesterol crystals within post-ischemic microglia makes this mechanism biologically plausible in stroke (14), but the complete sequence of crystal uptake, lysosomal membrane damage, NLRP3 assembly, and caspase-1 activation has not yet been directly demonstrated in post-stroke microglia. The crystal–lysosome–NLRP3 pathway should therefore be regarded as a strongly supported cross-model mechanism with emerging, but still incomplete, stroke-specific validation.

Other cellular stress pathways may provide additional points of convergence. K^+^ efflux and subsequent NEK7-dependent licensing are well-established components of canonical NLRP3 activation (34, 35), while mitochondrial dysfunction and mitochondrial reactive oxygen species are also closely associated with inflammasome activation during ischemic injury (5, 6, 36). However, there is currently insufficient evidence that cholesterol accumulation is itself a major direct driver of K^+^ efflux–NEK7 signaling in post-stroke microglia. Mitochondrial stress may provide a more plausible connection because lipid overload can coexist with disturbed mitochondrial function, and cholesterol mobilization has been shown to reduce microglial mitochondrial DNA leakage after stroke (14). Nevertheless, the available intervention data again identify STING, rather than NLRP3, as the more directly validated downstream sensor in this setting. These mechanisms are therefore better interpreted as shared cellular stress pathways through which cholesterol dysregulation may intersect with inflammasome susceptibility.

Metabolic and intracellular cholesterol-trafficking mechanisms provide further hypotheses but are supported mainly by cross-model evidence. PKM2-dependent glycolytic remodeling can promote NLRP3 and AIM2 inflammasome activation in inflammatory cells (31), whereas studies in atherosclerosis have shown that lysosomal cholesterol trafficking to the endoplasmic reticulum can promote CaMKII/JNK-dependent NLRP3 activation (33). These findings demonstrate that altered cellular metabolism and intracellular cholesterol distribution can regulate inflammasome activity, but neither pathway has yet been established as an obligatory link between cholesterol overload and NLRP3 activation in post-stroke microglia.

Oxysterol signaling further illustrates the context dependence of cholesterol-derived inflammatory regulation. In cerebral ischemia–reperfusion, 25-hydroxycholesterol has been reported to reduce brain injury through inhibition of STING signaling (12). Stroke-specific transcriptomic studies have also identified CH25H-expressing microglial subpopulations (37), indicating that oxysterol-generating pathways participate in post-ischemic microglial state remodeling. However, these observations do not demonstrate CH25H/25-hydroxycholesterol-dependent NLRP3 activation after stroke. By contrast, 25-hydroxycholesterol can promote NLRP3-associated inflammation in other neurological disease contexts (38). The direction of oxysterol signaling should therefore be considered dependent on molecular species, cellular context, concentration, and disease stage rather than uniformly pro-inflammatory.

Taken together, the available evidence favors a convergence model rather than a single linear cholesterol–NLRP3 pathway. Direct stroke studies support the coexistence of microglial cholesterol overload and NLRP3-related inflammatory changes, whereas crystal-induced lysosomal injury, intracellular cholesterol trafficking, and metabolic regulation of inflammasome activity remain partly dependent on mechanistic evidence from other inflammatory systems. General NLRP3 activation mechanisms, including ionic imbalance and mitochondrial stress, may intersect with these lipid-related processes but are not specific to cholesterol. Accordingly, cholesterol dysregulation should be considered a potential amplifier of the post-ischemic inflammasome-activating environment rather than an independently validated upstream cause of NLRP3 activation. The convergence of cholesterol-related lipid stress with NLRP3-associated signaling in post-stroke microglia is summarized in **Figure 2**.

**Figure 2:**
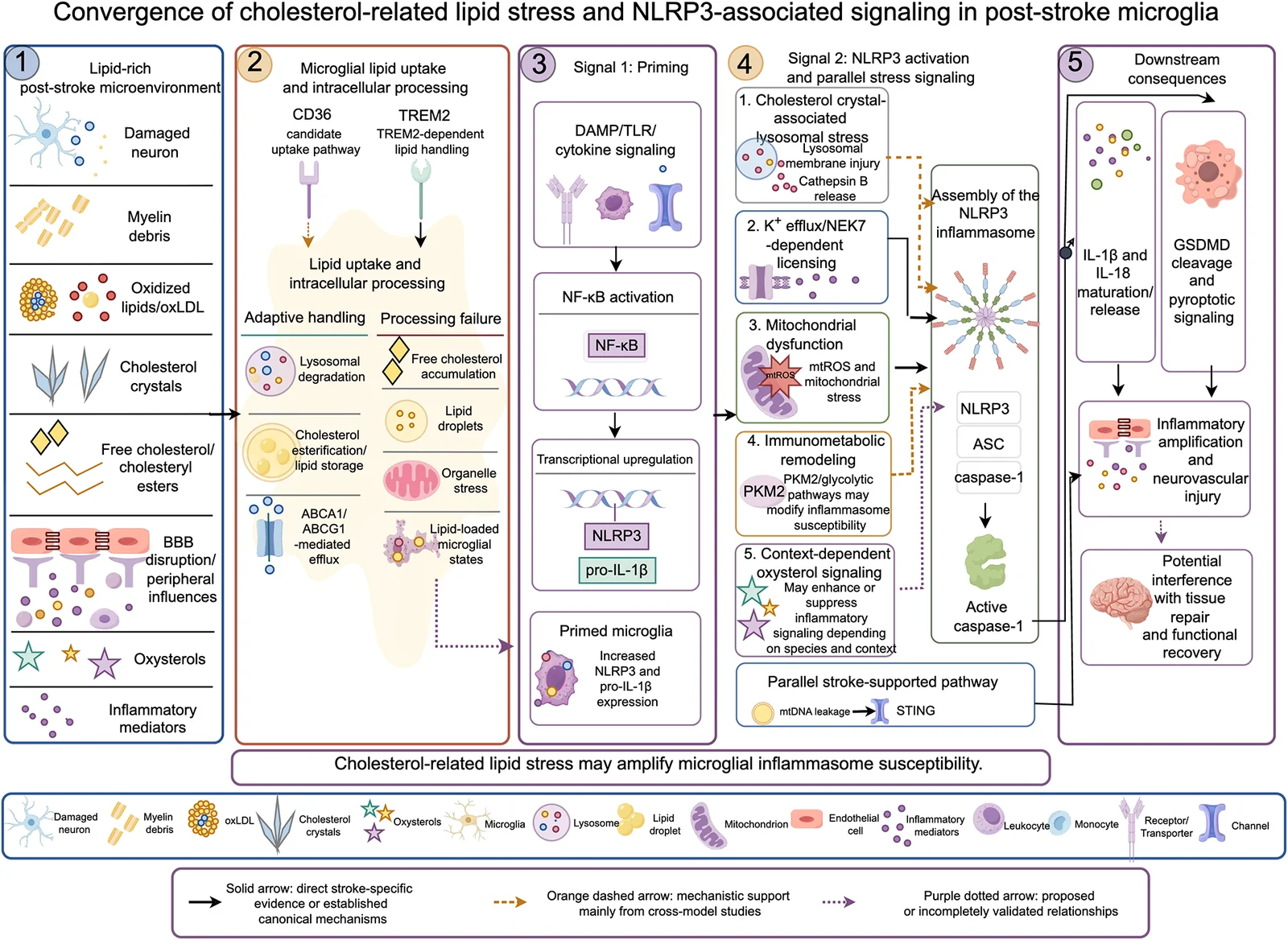
Convergence of cholesterol-related lipid stress and NLRP3-associated signaling in post-stroke microglia. Ischemic injury generates a lipid-rich microenvironment containing neuronal and myelin debris, free cholesterol, cholesteryl esters, cholesterol crystals, oxysterols, oxidized lipids, and inflammatory mediators. Microglia respond through lipid uptake, lysosomal degradation, cholesterol esterification and storage, intracellular trafficking, and ABCA1/ABCG1-mediated efflux. Persistent lipid exposure may exceed this processing capacity and promote free-cholesterol accumulation, lipid-droplet formation, organelle stress, and lipid-loaded microglial states. Canonical NLRP3 priming involves DAMP-, TLR-, and cytokine-related signaling with NF-κB-dependent induction of NLRP3 and pro-IL-1β, whereas activation can involve K^+^ efflux/NEK7-dependent licensing, mitochondrial stress, and lysosomal injury. Cholesterol crystal-associated lysosomal stress provides a plausible mechanism linking lipid overload to NLRP3 activation, but the complete crystal–lysosome–NLRP3 sequence remains incompletely demonstrated in post-stroke microglia. Immunometabolic remodeling and oxysterol signaling may further modify inflammasome susceptibility, although these relationships remain partly dependent on cross-model or context-specific evidence. Stroke-specific cholesterol-mobilization experiments also support mitochondrial DNA leakage–STING signaling as a parallel downstream pathway that has been more directly validated than an obligatory cholesterol–NLRP3 sequence. Activated caspase-1 promotes IL-1β/IL-18 maturation and release and GSDMD-mediated pyroptotic signaling, contributing to inflammatory amplification and potentially interfering with tissue repair and functional recovery. Black solid arrows indicate direct stroke-specific evidence or established canonical mechanisms; orange dashed arrows indicate mechanistic support derived mainly from cross-model studies; and purple dotted arrows indicate proposed or incompletely validated relationships. DAMPs, damage-associated molecular patterns; TLR, Toll-like receptor; NF-κB, nuclear factor κB; TREM2, triggering receptor expressed on myeloid cells 2; NEK7, NIMA-related kinase 7; mtROS, mitochondrial reactive oxygen species; PKM2, pyruvate kinase M2; STING, stimulator of interferon genes; ASC, apoptosis-associated speck-like protein containing a CARD; GSDMD, gasdermin D. By Figdraw.

### 4.3 Downstream effectors: IL-1β, IL-18, and pyroptosis

Once the NLRP3 inflammasome is assembled, activated caspase-1 processes pro-IL-1β and pro-IL-18 into their mature forms and cleaves gasdermin D (GSDMD), generating the pore-forming GSDMD N-terminal fragment (5–7). These events convert intracellular inflammasome activation into extracellular inflammatory signaling and, when GSDMD pore formation progresses sufficiently, pyroptotic membrane damage.

IL-1β is a major downstream mediator of inflammasome-associated inflammation after ischemic stroke and can amplify inflammatory signaling within the injured neurovascular environment (5, 6). IL-18 may also contribute to post-stroke immune activation, although its stroke-specific functions and relationship to neurological outcome are less well characterized than those of IL-1β (5, 6). The relative contribution of these cytokines is likely to vary according to the cellular source, lesion stage, and local inflammatory context.

GSDMD-mediated pyroptosis provides an additional mechanism for inflammatory amplification by promoting membrane permeabilization and the release of mature cytokines and other intracellular danger signals (7). Pyroptosis-related responses have been described in several neural and neurovascular cell populations after ischemic injury, although the dominant cellular contributors may differ across experimental models and disease stages (5–7). Importantly, IL-1β/IL-18 maturation and pyroptosis represent common downstream consequences of inflammasome activation rather than cholesterol-specific effects. The extent to which these downstream events contribute to acute tissue injury and later functional recovery is considered in Section 5.

### 4.4 Temporal, spatial, and cell-type heterogeneity of NLRP3-related responses after ischemic stroke

NLRP3-related inflammatory responses after ischemic stroke are temporally dynamic rather than uniform. Experimental studies have reported increased expression of NLRP3-related components and inflammasome downstream products during the acute and early subacute phases, although the magnitude and duration of these changes vary according to ischemia model, reperfusion status, lesion severity, and sampling time (5, 6). This temporal variability is important because early inflammasome activity develops within an environment dominated by ionic imbalance, mitochondrial injury, oxidative stress, and damage-associated molecular patterns, whereas later inflammatory responses increasingly coexist with debris clearance, lipid accumulation, and tissue remodeling.

The cellular distribution of NLRP3-related activity is similarly heterogeneous. Microglia are repeatedly implicated in inflammasome-associated responses after cerebral ischemia, and recent stroke-specific experiments have directly identified increased NLRP3 expression in Iba1+ microglia within peri-infarct tissue (14). However, microglia should not be assumed to be the exclusive or uniformly dominant cellular source. Infiltrating monocyte-derived macrophages, astrocytes, endothelial cells, neurons, and other neural cell populations may also express inflammasome-related components depending on disease stage and experimental context (4–6).

Single-cell, single-nucleus, and spatial transcriptomic studies further demonstrate substantial temporal and regional heterogeneity among post-stroke glial and myeloid populations (25–27). These datasets are useful for identifying cell states and inflammatory or metabolic gene programs, but transcript abundance alone does not establish functional inflammasome assembly, caspase-1 activation, or cytokine processing. Accordingly, transcriptomic evidence should be used to define the cellular context in which NLRP3 activation may occur rather than as direct proof of cell-specific inflammasome activity.

This distinction is particularly relevant to cholesterol-loaded microglia. Chronic and subacute stroke studies identify lipid-rich or foam-like microglial states that persist beyond the early injury period (10, 14), but current evidence does not establish that these later lipid-loaded states are necessarily accompanied by proportionally greater NLRP3 activation. The relative contribution of classical ischemic stress signals versus cholesterol-related metabolic stress is therefore likely to change over time, and direct longitudinal studies combining lipid measurements with cell-specific inflammasome activation assays remain needed.

### 4.5 Summary

Current evidence supports NLRP3 inflammasome activation as an important component of the inflammatory response to ischemic stroke, with ischemia–reperfusion providing overlapping signals for inflammasome priming and activation. Microglia are among the cell populations involved in these responses, and recent stroke-specific evidence demonstrates that cholesterol-loaded microglial states can coexist with increased NLRP3 expression (5, 6, 14). Once activated, the NLRP3–caspase-1 pathway promotes IL-1β and IL-18 maturation and GSDMD-mediated pyroptotic signaling, providing a mechanism through which intracellular stress can amplify inflammation within the injured brain (5–7).

However, the evidence does not yet establish a single cholesterol-specific pathway that is required for NLRP3 activation after stroke. Direct stroke studies support microglial cholesterol accumulation, cholesterol crystal formation, and NLRP3-associated inflammatory changes, but the detailed mechanisms linking these events remain only partly resolved (14). Crystal-induced lysosomal injury, PKM2-dependent metabolic regulation, intracellular cholesterol trafficking, and other mechanisms provide biologically plausible routes of convergence, but several are supported primarily by macrophage, atherosclerosis, or other inflammatory models (13, 31, 33). NLRP3 activation after stroke should therefore be understood as a context- and stage-dependent convergence of ischemic, metabolic, and organelle stress signals, with cholesterol dysregulation acting as a potential amplifier rather than an independently validated upstream cause. The functional consequences of this inflammatory response for acute injury and subsequent neurological recovery are considered in Section 5.

## 5 Consequences for neuroinflammation and functional recovery

The biological relevance of the proposed cholesterol–microglia–NLRP3 framework ultimately depends on whether the associated inflammatory responses influence tissue injury and neurological recovery after ischemic stroke. These consequences are likely to vary over time. During the acute phase, excessive inflammasome-associated signaling can contribute to inflammatory amplification and neurovascular injury, whereas during the subacute and chronic phases, persistent inflammatory and lipid-metabolic disturbances may interfere with processes required for tissue repair, including synaptic remodeling, white matter restoration, and neurovascular recovery (1, 5–7, 10).

Importantly, evidence for these downstream consequences is stronger for NLRP3-associated inflammation in general than for a complete cholesterol-driven NLRP3 pathway. Direct stroke studies support relationships among inflammasome activity, inflammatory injury, and neurological outcomes, while cholesterol-loaded microglial states have been linked particularly to persistent inflammation and impaired repair (10, 14). This section therefore distinguishes established consequences of post-stroke inflammasome-related inflammation from recovery effects that remain only indirectly connected to cholesterol dysregulation.

### 5.1 Acute phase: inflammasome-associated neurovascular injury and infarct evolution

During the acute phase of ischemic stroke, inflammasome-associated inflammation develops within a neurovascular environment already exposed to energy failure, oxidative stress, blood–brain barrier disruption, and cell death. NLRP3 activation can amplify this response through caspase-1-dependent cytokine maturation and GSDMD-mediated pyroptotic signaling (5–7). The resulting release of IL-1β and other inflammatory mediators can reinforce local immune activation and contribute to endothelial dysfunction, leukocyte recruitment, edema, and secondary injury within vulnerable peri-infarct tissue (1, 5, 6).

Experimental stroke studies support a relationship between enhanced NLRP3-related signaling and greater acute brain injury. Increased expression or activation of NLRP3, ASC, caspase-1, IL-1β, and pyroptosis-associated proteins has been reported after cerebral ischemia, while interventions that attenuate inflammasome-related signaling are frequently accompanied by reductions in neuroinflammation and ischemic injury (5–7, 39). For example, inhibition of JAK2/STAT3 signaling in experimental ischemic stroke reduced NLRP3 inflammasome activation and was associated with reduced inflammatory injury and improved neurological outcomes (39). Because JAK2/STAT3 regulates multiple inflammatory pathways, however, this study supports the pathological relevance of NLRP3-associated signaling but does not by itself establish NLRP3-specific causality.

The contribution of cholesterol dysregulation to these acute effects is less certain. Recent stroke-specific evidence shows that reducing microglial cholesterol burden can decrease inflammatory changes and improve infarct-related and neurological outcomes (14). However, the strongest mechanistic evidence in that study implicated mitochondrial DNA leakage and STING signaling rather than demonstrating that NLRP3 was required for the protective effect of cholesterol mobilization (14). Thus, acute neurovascular injury is well supported as a consequence of excessive post-stroke inflammation, whereas the proportion of this injury specifically attributable to a cholesterol–microglial NLRP3 mechanism remains unresolved.

### 5.2 Subacute and chronic phases: inflammation, tissue repair, and functional recovery

As ischemic stroke progresses beyond the acute injury phase, the biological consequences of inflammation increasingly depend on whether immune responses resolve appropriately and support tissue remodeling. Persistent inflammasome-associated signaling may interfere with recovery-related processes, but post-stroke microglial responses are not uniformly detrimental. Microglia also participate in debris clearance, vascular remodeling, and white matter repair, and distinct repair-associated states emerge during the subacute and chronic phases (25, 26, 40, 41). Functional outcome therefore depends not simply on the magnitude of microglial activation, but on the timing, cellular state, and metabolic context in which that activation occurs.

Evidence linking NLRP3-related inflammation to delayed neurological dysfunction is strongest in experimental models of cognitive and neurovascular recovery. In a model of ischemia complicated by diabetes, pharmacological inhibition of NLRP3 with MCC950 improved cognitive performance and vasoneuronal remodeling (42). This finding supports a contribution of persistent NLRP3 activity to delayed functional impairment, although the metabolic comorbidity and specific experimental setting limit direct generalization to all forms of ischemic stroke. More broadly, inflammatory cytokines and oxidative stress can influence synaptic and vascular remodeling after ischemia, but evidence directly assigning post-stroke synaptic dysfunction to a cholesterol-dependent microglial NLRP3 mechanism remains limited.

White matter repair provides a particularly informative example of the context-dependent role of microglia. Experimental stroke studies have identified microglial populations that actively support remyelination and tissue repair. Treg cell-derived osteopontin promotes microglia-mediated white matter repair after ischemic stroke (40), while CD11c^+^ microglia have independently been shown to facilitate white matter restoration (41). TREM2-related microglial signaling is also associated with recovery: physical exercise promotes neurological improvement and white matter repair through a TREM2-dependent microglial mechanism in experimental stroke (43). These observations argue against a simple model in which sustained microglial activation is uniformly detrimental and instead support the existence of functionally distinct inflammatory, lipid-processing, and repair-associated states.

Cholesterol metabolism becomes particularly relevant when microglial lipid processing fails to resolve during the later stages of injury. Persistent cholesterol accumulation and stroke-associated foamy microglial states have been linked to chronic neuroinflammation and impaired neurorestoration, whereas enhancement of cholesterol clearance reduces lipid overload and promotes white matter repair and functional recovery (10). Consistent with this concept, recent stroke-specific evidence shows that pharmacological reduction of microglial cholesterol burden decreases lipid-loaded microglial states, preserves white matter integrity, and improves motor outcomes (14). TREM2-dependent regulation of cholesterol turnover and lipid droplet formation provides an additional connection between microglial lipid-processing capacity and the post-stroke repair environment (22).

These findings support a relationship between persistent microglial lipid dysregulation and impaired recovery, but they do not establish that NLRP3 is the obligatory mediator of this effect. The protective consequences of improved cholesterol handling can involve multiple inflammatory and metabolic pathways; in this cholesterol-mobilization study, STING signaling was more directly validated than NLRP3 as a downstream mediator of cholesterol-related injury (14). Similarly, TREM2-dependent white matter repair and repair-associated microglial populations can influence recovery without requiring a demonstrated NLRP3 mechanism. The current evidence therefore supports partially overlapping links among cholesterol handling, microglial state, inflammasome-associated inflammation, and tissue repair rather than a single linear pathway from cholesterol accumulation to NLRP3-dependent recovery failure.

### 5.3 Human and translational evidence for recovery-related outcomes

Human evidence linking cholesterol dysregulation, microglial inflammasome activity, and neurological recovery after ischemic stroke remains substantially less developed than the experimental literature. Within the clinical evidence identified for this review, no study directly evaluates brain or microglial cholesterol accumulation, cell-specific NLRP3 activation, and longitudinal functional recovery within the same patient cohort. Available studies instead provide indirect evidence through circulating lipid-related measures, thrombus lipid composition, stroke severity, and short-term functional outcomes.

One prospective clinical study of patients with acute ischemic stroke found that reduced HDL cholesterol efflux capacity and altered HDL composition were associated with greater neurological severity and functional dependency at discharge (18). These findings suggest that functional measures of peripheral cholesterol handling may carry prognostic information beyond HDL cholesterol concentration alone. However, HDL cholesterol efflux capacity reflects systemic lipoprotein function and does not directly measure cholesterol processing within the ischemic brain, microglial lipid burden, or NLRP3 activity. Moreover, the reported outcome was assessed during hospitalization rather than over long-term neurological recovery.

Lipidomic analysis of paired thrombi and plasma from patients undergoing endovascular thrombectomy provides additional evidence that lipid alterations may be compartment specific (19). Distinct lipid signatures were identified in thrombi across stroke etiologies, whereas significant corresponding differences were not detected in plasma (19). This negative plasma finding is relevant to biomarker development because it cautions against assuming that circulating lipid profiles faithfully reflect lipid remodeling within the thrombus or injured brain. However, this study was designed primarily to examine stroke etiology rather than post-stroke recovery and therefore does not establish a relationship between these lipid signatures and microglial inflammasome activity or long-term functional outcome.

These limitations define an important translational gap. Future clinical studies will need to integrate lipid-related biomarkers with inflammatory measurements, imaging indicators of tissue injury and repair, and longitudinal functional outcomes. Where feasible, cerebrospinal fluid or extracellular-vesicle markers may provide additional information closer to the central nervous system than circulating lipid concentrations alone. Outcome assessment should extend beyond early neurological scores to include modified Rankin Scale trajectories, motor and cognitive recovery, white matter integrity, and other measures of durable neurological repair. Until such multidimensional studies are available, the human evidence should be interpreted as supportive of a relationship between lipid metabolism and stroke outcome, but not as clinical validation of a cholesterol–microglia–NLRP3 causal pathway.

### 5.4 Evidence synthesis and unresolved links

Taken together, current evidence indicates that inflammasome-associated inflammation can worsen ischemic injury and may also influence subsequent neurological recovery, but its biological effects are strongly dependent on disease stage and microglial state. Pharmacological inhibition of NLRP3 has improved delayed cognitive and vasoneuronal outcomes in selected experimental settings (42), whereas distinct repair-associated microglial populations and TREM2-dependent responses can promote white matter restoration and functional recovery (40, 41, 43). In parallel, persistent microglial cholesterol accumulation has been linked to chronic neuroinflammation and impaired neurorestoration, while interventions that reduce cholesterol burden can improve white matter integrity and neurological function (10, 14). These findings support a recovery-oriented model in which lipid-processing capacity, inflammatory signaling, and microglial state jointly influence tissue repair rather than a simple model in which all microglial activation or inflammasome-related responses are uniformly detrimental.

However, the mechanistic overlap among cholesterol dysregulation, microglial NLRP3 activation, and functional recovery remains incompletely resolved. Stroke-specific studies directly support cholesterol-loaded microglial states, inflammasome-related inflammatory changes, and recovery effects of cholesterol-modifying interventions, but they do not establish NLRP3 as the obligatory mediator linking lipid dysregulation to failed repair (10, 14). Human evidence is even more indirect: peripheral cholesterol efflux measures are associated with acute stroke severity and short-term functional outcome, whereas thrombus and plasma lipidomics demonstrate compartment-specific lipid signatures and a lack of corresponding significant plasma differences across stroke etiologies (18, 19). Neither approach directly measures brain microglial cholesterol burden, cell-specific inflammasome activation, or long-term neurological recovery. Future studies should therefore prioritize longitudinal and cell-resolved approaches that integrate lipid state, inflammasome activity, tissue-repair markers, and functional outcomes across defined post-stroke time windows. Until such evidence is available, the cholesterol–microglia–NLRP3 relationship is best regarded as a proposed, partially overlapping mechanistic framework rather than a validated linear causal pathway. The evidence linking cholesterol metabolism, microglia, NLRP3 activation, white matter repair, and functional recovery is summarized in **Table 1**.

**Table 1:** Evidence-stratified summary of the proposed cholesterol–microglia–NLRP3 framework and recovery-related outcomes after ischemic stroke.

Evidence domain (refs)	Representative findings	Main model/context	Recovery-related relevance	Evidence category/key limitation
Post-ischemic lipid remodeling and cholesterol-loaded microglia (10, 11, 14)	Experimental stroke produces dynamic remodeling of cholesterol-related lipids, including cholesteryl ester and lipid-droplet accumulation. Persistent free cholesterol, cholesterol crystals, and lipid-loaded/foamy microglial states have been demonstrated after ischemic injury. Cholesterol clearance or mobilization reduces microglial lipid burden and inflammatory changes.	MCAO, dMCAO and tMCAO; lipidomics, scRNA-seq, histology and pharmacological intervention.	CYP46A1-mediated cholesterol clearance and pharmacological cholesterol mobilization are associated with improved white matter integrity and neurological recovery.	Level I: direct stroke-specific evidence. Strong evidence for post-stroke microglial cholesterol overload; NLRP3 has not been established as the obligatory downstream mediator of the recovery effects.
Cholesterol crystals and lysosomal–NLRP3 convergence (10, 13, 14)	Cholesterol crystals accumulate in post-ischemic microglia and coexist with increased NLRP3 expression. Crystal uptake can induce lysosomal injury and NLRP3 activation in macrophage and atherosclerosis models.	Experimental stroke; macrophage and atherosclerosis models.	Persistent crystal-containing lipid-loaded states may contribute to chronic inflammatory stress, but recovery effects cannot yet be assigned specifically to crystal-induced NLRP3 activation.	Level I for crystal accumulation/NLRP3 association; Level III for the detailed lysosomal mechanism. The complete crystal uptake → lysosomal injury → NLRP3 assembly → caspase-1 sequence has not been directly demonstrated in post-stroke microglia.
Oxysterol signaling (12, 15–17, 37, 38)	Oxysterol effects are heterogeneous. In cerebral ischemia–reperfusion, 25-HC can reduce injury through STING inhibition, whereas CH25H/25-HC and 7-KC can promote inflammatory signaling in other neurological or lipid-rich inflammatory settings.	Cerebral ischemia–reperfusion; stroke transcriptomics; epilepsy, X-linked adrenoleukodystrophy and retinal microglial models.	Oxysterols may influence injury or recovery differently according to molecular species, concentration, cell type and disease stage.	Mixed Level I and Level III evidence. Direct stroke evidence includes a protective 25-HC effect and CH25H-expressing microglial states; pro-NLRP3 effects are supported mainly by cross-model studies.
Microglial lipid-handling capacity: TREM2, SREBP2 and lipid droplets (10, 11, 22–24)	TREM2 deficiency impairs phagocytosis and cholesterol hydrolysis/efflux and increases cholesteryl ester and lipid-droplet accumulation. S1P–TREM2 signaling promotes phagocytosis. SREBP2-associated lipid and autophagy dysregulation contributes to inflammatory microglial states, whereas SREBP2 suppression reduces lipid burden and inflammatory signaling.	Experimental ischemic stroke, MCAO/OGD-R and microglia–neuron co-culture models.	Effective lipid processing may support debris clearance and recovery, whereas persistent lipid-processing failure is associated with tissue injury and poorer neurological outcome.	Level I: direct stroke-specific evidence. Strong support for lipid-processing mechanisms, but direct TREM2→NLRP3 or SREBP2→NLRP3 causality remains unproven.
Microglial state heterogeneity and immunometabolic remodeling (25–32)	Single-cell, single-nucleus and spatial studies demonstrate temporally and regionally heterogeneous post-stroke myeloid states. Mitochondrial fitness influences inflammatory behavior, whereas glycolytic/PKM2-related mechanisms provide potential routes linking metabolic stress with inflammasome susceptibility.	Experimental stroke transcriptomics and metabolic studies; macrophage and other inflammatory models.	Metabolic state may influence whether microglia sustain inflammation or transition toward repair-supportive functions.	Level I for post-stroke cellular heterogeneity and selected metabolic regulators; Level III for several detailed glycolytic mechanisms. A cholesterol-specific metabolic sequence leading to NLRP3 activation has not been established.
NLRP3-associated inflammation and pyroptotic signaling (5–7, 39, 42)	Ischemic injury provides overlapping signals for NLRP3 priming and activation. NLRP3–caspase-1 signaling promotes IL-1β/IL-18 maturation and GSDMD-mediated pyroptosis. Experimental attenuation of NLRP3-associated signaling can reduce inflammatory injury.	Experimental ischemic stroke; pharmacological intervention; diabetic ischemia model.	Excessive inflammasome-associated signaling contributes to acute injury and may influence delayed cognitive and neurovascular outcomes.	Level I: stroke-specific experimental evidence for NLRP3-associated injury and intervention. JAK2/STAT3 inhibition is pleiotropic, and MCC950 recovery evidence is derived from a metabolically comorbid model; these effects are not cholesterol-specific.
Repair-supportive versus maladaptive myeloid states (10, 20, 40, 41, 43)	Post-stroke myeloid responses are not uniformly detrimental. Repair-associated macrophages, osteopontin-responsive microglia, CD11c+ microglia and TREM2-dependent responses can support white matter, vascular and neurological recovery. Persistent cholesterol-loaded microglia, by contrast, are associated with chronic inflammation and impaired neurorestoration.	Experimental stroke and rehabilitation studies.	Provides direct evidence that recovery depends on myeloid cell state rather than on generalized suppression of microglial activation.	Level I: direct stroke-specific recovery evidence. Strong counter-evidence to a uniformly pro-inflammatory microglial model; the complete cholesterol → NLRP3 → failed repair sequence remains unvalidated.
Human and translational evidence (18, 19)	Reduced HDL cholesterol efflux capacity is associated with greater stroke severity and functional dependency at discharge. Thrombus lipidomics identifies etiology-associated lipid differences, whereas corresponding significant plasma differences were not detected.	Patients with acute ischemic stroke; prospective clinical study and paired thrombus/plasma lipidomics.	Supports a clinical relationship between systemic lipid handling and stroke outcome, while highlighting compartment-specific biomarker limitations.	Level II: human stroke-associated but mechanistically indirect evidence. Neither study directly measures brain microglial cholesterol burden, cell-specific NLRP3 activity or long-term recovery.

## 6 Therapeutic strategies and future directions

The therapeutic implications of the proposed cholesterol–microglia–NLRP3 framework should be interpreted according to the level of evidence supporting each intervention point. Within the literature considered in this review, no therapy has been clinically validated to specifically modify microglial cholesterol dysregulation and NLRP3 activation as an integrated mechanism after ischemic stroke. Current therapeutic evidence instead spans distinct domains: clinically established systemic lipid management for secondary vascular prevention, stroke-specific preclinical interventions that modify brain or microglial cholesterol handling, and experimental approaches that suppress inflammasome-associated inflammatory signaling (10, 14, 22, 24, 39, 42, 44).

These therapeutic domains should not be considered interchangeable. Reduction of systemic vascular lipid risk does not necessarily normalize cholesterol handling within the injured brain, whereas experimental strategies that mobilize microglial cholesterol or modify lipid-processing pathways remain largely preclinical. Similarly, inhibition of NLRP3 can reduce inflammatory amplification without directly correcting the upstream lipid burden. A more realistic therapeutic framework is therefore stage dependent: vascular risk management provides a clinical foundation, early excessive inflammatory signaling may require selective restraint, and later phases may benefit from interventions that restore lipid processing and support white matter and functional recovery.

### 6.1 Systemic lipid management: established vascular benefit but limited relevance to brain cholesterol dysregulation

Lipid-lowering therapy is an established component of secondary vascular prevention after ischemic stroke in appropriately selected patients. Current stroke-prevention guidelines recommend intensive lipid management, including statin-based therapy and additional lipid-lowering agents when indicated, to reduce recurrent vascular events (44). This clinical benefit, however, should be distinguished from treatment of post-ischemic brain cholesterol dysregulation. Evidence that systemic lipid lowering directly normalizes microglial cholesterol accumulation, lipid droplet formation, or inflammasome activity within the injured brain is currently lacking.

Human observational studies further illustrate this distinction. Reduced HDL cholesterol efflux capacity and altered HDL composition have been associated with greater acute stroke severity and functional dependency at discharge (18), suggesting that systemic lipoprotein function may provide clinically relevant prognostic information. However, these measurements do not directly reflect cholesterol processing within post-stroke microglia. Similarly, lipidomic analysis of paired thrombi and plasma from patients undergoing endovascular thrombectomy identified stroke-etiology-associated lipid differences in thrombi but no corresponding significant differences in plasma (19). These findings highlight the compartment-specific nature of lipid remodeling and caution against using peripheral lipid measures as direct surrogates for the lipid environment of the ischemic brain.

This distinction is also biologically consistent with the relative compartmentalization of brain cholesterol metabolism. Experimental stroke studies demonstrate substantial local lipid remodeling, microglial lipid droplet formation, persistent cholesterol accumulation, and cholesterol-loaded microglial states after ischemic injury (10, 11, 14). Such changes arise in the context of local membrane and myelin damage and altered cellular cholesterol processing and therefore cannot be assumed to normalize simply through correction of circulating lipid concentrations.

Systemic lipid management should consequently be regarded as an evidence-based strategy for vascular risk reduction rather than as a validated treatment for the proposed brain cholesterol–microglia–NLRP3 framework. Future therapeutic studies should distinguish endpoints related to recurrent vascular risk from those intended to demonstrate modification of brain lipid handling, neuroinflammation, white matter repair, or neurological recovery. The latter will require interventions and biomarkers capable of interrogating cholesterol metabolism within the post-ischemic central nervous system more directly.

### 6.2 Targeting post-stroke microglial cholesterol handling and lipid burden

Strategies that directly reduce the post-ischemic cholesterol burden or restore intracellular lipid-processing capacity may be particularly relevant for persistent neuroinflammation and recovery impairment. Among the therapeutic approaches considered here, the strongest stroke-specific preclinical evidence currently comes from interventions targeting cholesterol clearance or mobilization. In chronic experimental stroke, genetic or pharmacological enhancement of CYP46A1-mediated cholesterol turnover reduced microglial cholesterol overload and persistent neuroinflammation and promoted white matter repair and functional recovery (10). More recently, pharmacological cholesterol mobilization with 2-hydroxypropyl-β-cyclodextrin (HβCD) reduced free cholesterol, cholesterol-associated lipid droplets, and inflammatory microglial states and improved infarct-related, white matter, and motor outcomes across experimental stroke models (14). Together, these studies provide direct evidence that excessive post-stroke cholesterol burden is therapeutically modifiable, although neither intervention has yet been established for this purpose in patients with ischemic stroke.

TREM2 represents another important regulatory node, but its therapeutic evidence should be distinguished from that of direct cholesterol-mobilizing interventions. In experimental ischemic conditions, TREM2 deficiency impaired microglial phagocytosis, promoted cholesteryl ester and lipid droplet accumulation, and reduced expression of genes involved in cholesterol hydrolysis, trafficking, and efflux (22). In a separate stroke study, sphingosine-1-phosphate acted as a TREM2 ligand and enhanced microglial phagocytosis in a TREM2-dependent manner, with corresponding protection against ischemic brain injury (23). These studies support TREM2-dependent lipid handling as a biologically relevant therapeutic target, but direct pharmacological TREM2 agonism has not yet been comparably validated in ischemic stroke. TREM2 should therefore be regarded as a promising regulatory target rather than an established stroke therapy.

Intracellular cholesterol synthesis and processing may provide an additional intervention point. Recent stroke-specific evidence implicates sterol regulatory element-binding protein 2 (SREBP2) in cholesterol-related lipid accumulation, autophagy-associated dysregulation, and microglial inflammatory responses after cerebral ischemia (24). Experimental suppression of SREBP2 reduced cholesterol-related lipid accumulation and inflammatory signaling, improved neuronal survival in microglia–neuron co-culture experiments, and improved neurological function after MCAO (24). These findings extend therapeutic consideration beyond lipid uptake and efflux to the intracellular programs that regulate cholesterol synthesis and processing. However, the study remains preclinical, and its effects should not be interpreted as evidence for direct suppression of NLRP3 because the SREBP2–autophagy study did not establish NLRP3 as the required downstream mediator.

Other cholesterol-efflux pathways, including LXR-regulated ABCA1/ABCG1 signaling, remain mechanistically attractive because they regulate cellular cholesterol export and lipoprotein remodeling (8). However, the stroke-specific therapeutic evidence for direct LXR–ABCA1/ABCG1 manipulation is currently less developed than that for CYP46A1-mediated cholesterol clearance, HβCD-mediated cholesterol mobilization, or the experimental manipulation of TREM2 and SREBP2. Broad activation of lipid-regulatory pathways may also affect multiple cell populations and systemic metabolic processes. Future studies should therefore determine whether these approaches can selectively normalize pathological lipid-loaded microglial states without disrupting lipid-dependent debris clearance or repair-supportive microglial functions.

Overall, upstream correction of post-stroke cholesterol handling is supported by increasingly direct experimental evidence, but the therapeutic maturity of individual targets differs substantially. CYP46A1 modulation and cholesterol mobilization currently provide the clearest proof-of-concept that reducing brain or microglial cholesterol burden can improve recovery-related outcomes, whereas TREM2, SREBP2, and LXR-related pathways represent targets at different stages of preclinical validation. None has yet demonstrated clinical efficacy as a brain cholesterol-directed therapy after ischemic stroke. These upstream approaches are mechanistically distinct from direct NLRP3 inhibition, which suppresses inflammatory amplification without necessarily correcting the underlying lipid burden and is considered in Section 6.3.

### 6.3 Targeting NLRP3 and inflammasome-associated inflammatory amplification

Direct inhibition of NLRP3 represents a downstream strategy for limiting inflammasome-associated inflammatory amplification after ischemic stroke. Among currently available experimental approaches, MCC950 provides one of the clearest pharmacological tools for selectively suppressing NLRP3 activity (45). In an ischemic model complicated by diabetes, MCC950 improved cognitive performance and vasoneuronal remodeling, supporting the possibility that NLRP3 inhibition can influence recovery-related outcomes in addition to acute inflammatory injury (42). However, the comorbid experimental setting limits generalization to the broader ischemic stroke population, and within the literature considered in this review, NLRP3-directed pharmacotherapy remains preclinical.

Other stroke studies support the therapeutic relevance of inflammasome-associated signaling but provide less specific evidence for direct NLRP3 targeting. In experimental ischemic stroke, inhibition of the JAK2/STAT3 pathway reduced NLRP3 inflammasome activation, neuroinflammation, and ischemic injury (39). Because JAK2/STAT3 regulates multiple inflammatory and survival pathways, these findings support NLRP3 as one component of a broader inflammatory response rather than demonstrating that the therapeutic effect is mediated exclusively through NLRP3. Such upstream or pleiotropic interventions should therefore be distinguished from direct inflammasome inhibition.

Importantly, NLRP3 inhibition addresses inflammatory amplification downstream of cellular stress but does not directly correct the lipid-processing abnormalities that may sustain that stress. Blocking NLRP3 would not be expected, by itself, to remove accumulated cholesterol, mobilize lipid droplets, restore cholesterol efflux, or normalize lipid-loaded microglial states (10, 14). Conversely, cholesterol-directed interventions can improve inflammatory and neurological outcomes without requiring NLRP3 to be the sole downstream mediator; recent cholesterol-mobilization experiments more directly implicated mitochondrial DNA leakage and STING signaling (14). These observations argue against treating upstream lipid modulation and downstream inflammasome inhibition as mechanistically equivalent strategies.

Therapeutic timing is also likely to be critical. Excessive inflammasome-associated signaling during acute and early subacute injury may represent an appropriate target for temporary suppression, whereas prolonged or poorly timed inhibition could theoretically interfere with immune functions required for debris clearance, inflammatory resolution, or tissue remodeling. This concern is particularly relevant because post-stroke microglial populations include both inflammatory and repair-supportive states (40, 41, 43). Future studies should therefore define the optimal treatment window, duration, cellular target, and recovery endpoints for NLRP3-directed therapy rather than evaluating efficacy solely by acute infarct volume or inflammatory-marker reduction.

Overall, NLRP3 remains a plausible downstream therapeutic target within the proposed framework, but current evidence does not support its inhibition as a substitute for correcting upstream cholesterol dysregulation. A stage-specific strategy in which excessive inflammasome signaling is selectively restrained while lipid clearance and repair-supportive microglial functions are preserved may be more biologically appropriate than sustained, nonspecific suppression of post-stroke inflammation.

### 6.4 Stage-specific and recovery-oriented combination strategies

The temporal evolution of ischemic stroke argues against a uniform therapeutic strategy across all stages of injury and recovery. During the acute and early subacute phases, excessive inflammasome-associated signaling may contribute to inflammatory amplification and secondary tissue injury, providing a rationale for appropriately timed suppression of NLRP3-related responses (5–7, 39). By contrast, during the subacute and chronic phases, persistent lipid accumulation, impaired debris processing, and maladaptive microglial states become increasingly relevant to white matter and functional recovery (10, 14, 22). These differences suggest that the therapeutic priority may shift over time from limiting excessive inflammatory amplification toward restoring lipid-processing capacity and supporting tissue repair.

Recovery-oriented interventions may complement this stage-specific approach. Exercise provides a useful experimental example because its effects extend beyond acute neuroprotection. In experimental stroke, physical exercise promoted neurological recovery through TREM2-dependent microglial support of white matter repair (43), while a separate study reported that exercise reduced ischemia–reperfusion injury in association with attenuation of inflammasome-related pyroptosis (46). These findings indicate that rehabilitation can interact with both inflammatory and repair-related pathways, although they do not establish that exercise specifically modifies a cholesterol–NLRP3 mechanism.

The available evidence therefore supports the rationale for sequential or complementary interventions rather than simultaneous broad suppression of all inflammatory and metabolic responses. One conceptual strategy would be to limit excessive inflammasome-associated signaling during early injury, enhance cholesterol clearance or microglial lipid-processing capacity as the lesion enters the remodeling phase, and combine these approaches with rehabilitation that supports white matter and network recovery. However, this sequence remains a therapeutic hypothesis rather than a validated treatment regimen. No study identified in this review has directly compared or combined cholesterol-directed therapy, selective NLRP3 inhibition, and rehabilitation across defined post-stroke time windows.

Future preclinical studies should therefore test treatment timing and combination strategies prospectively rather than inferring them from interventions examined separately. Such studies should include both acute injury measures and longer-term endpoints, including motor and cognitive function, white matter integrity, remyelination, and neurovascular remodeling. Particular attention should be given to whether early anti-inflammatory treatment alters subsequent microglial lipid handling or repair-associated states and whether later cholesterol-directed interventions enhance, rather than interfere with, endogenous debris clearance and remodeling. This stage-specific design would provide a stronger basis for determining whether upstream metabolic intervention and downstream inflammasome modulation are complementary, redundant, or potentially antagonistic.

The therapeutic strategies, candidate targets, intervention windows, recovery-related relevance, and major translational limitations discussed above are summarized in **Table 2**. The stage-specific therapeutic opportunities and their relative levels of evidence are summarized in **Figure 3**.

**Table 2:** Evidence-stratified therapeutic strategies relevant to the proposed cholesterol–microglia–NLRP3 framework after ischemic stroke.

Therapeutic strategy/target (refs)	Representative evidence and proposed action	Potential intervention stage	Recovery-related relevance	Evidence status/major limitation
Systemic lipid management (18, 19, 44)	Guideline-based lipid management, including statin-based therapy and additional lipid-lowering agents when indicated, reduces recurrent vascular risk. Peripheral HDL cholesterol efflux capacity is associated with stroke severity and short-term outcome, whereas thrombus and plasma lipidomics reveal compartment-specific differences.	Secondary prevention; long-term vascular risk management.	Reduces recurrent vascular events but is not established to normalize cholesterol-loaded microglia or brain inflammasome activity.	Established clinical secondary-prevention strategy, but not a validated brain cholesterol-directed therapy. Peripheral lipid measures should not be treated as surrogates for post-stroke microglial cholesterol metabolism.
CYP46A1-mediated cholesterol clearance and HβCD-mediated cholesterol mobilization (10, 14)	Enhancement of CYP46A1-mediated cholesterol turnover reduces persistent microglial cholesterol overload and chronic neuroinflammation. HβCD reduces free cholesterol, cholesterol crystals and lipid-droplet burden in post-stroke microglia and improves neurological outcomes.	Particularly relevant to subacute/chronic lipid accumulation and remodeling; HβCD has also been tested early after experimental stroke.	Associated with improved white matter integrity, reduced inflammatory burden and better neurological recovery.	Direct stroke-specific preclinical intervention evidence. Provides strong proof-of-concept for targeting cholesterol burden, but clinical efficacy is untested; HβCD-related protection cannot be attributed specifically to NLRP3 because STING was more directly validated downstream.
TREM2-dependent lipid handling (22, 23)	TREM2 deficiency impairs phagocytosis, cholesterol hydrolysis/efflux and lipid-droplet turnover. S1P–TREM2 signaling enhances microglial phagocytosis and reduces ischemic injury.	Subacute/remodeling phase, when debris clearance and lipid processing become prominent.	May preserve adaptive microglial lipid processing and support a repair-permissive environment.	Direct stroke-specific biological evidence with emerging therapeutic relevance. Selective pharmacological TREM2 agonism has not been comparably validated in ischemic stroke, and TREM2→NLRP3 causality is unproven.
SREBP2–autophagy-associated lipid processing (24)	Experimental suppression of microglial SREBP2 reduces cholesterol-related lipid accumulation, excessive autophagy-associated responses and inflammatory signaling and improves neuronal survival and neurological outcome.	Potentially subacute, when intracellular lipid accumulation and metabolic remodeling are established.	May reduce maladaptive lipid storage and inflammatory microglial states and improve neurological recovery.	Direct stroke-specific preclinical evidence. Findings require independent validation; NLRP3 was not established as the required downstream mediator.
LXR–ABCA1/ABCG1 cholesterol-efflux pathways (8)	LXR-regulated ABCA1/ABCG1 signaling promotes cellular cholesterol efflux and ApoE-dependent lipid transport and is therefore mechanistically attractive as a strategy for improving cholesterol handling.	Candidate strategy for subacute/chronic lipid-processing dysfunction.	Could theoretically facilitate removal or redistribution of excess cellular cholesterol and reduce lipid stress.	Mechanistic candidate with limited stroke-specific therapeutic validation. Broad pathway activation may affect multiple CNS and peripheral cell populations; efficacy in post-stroke lipid-loaded microglia remains unproven.
Direct NLRP3 inhibition with MCC950 (42, 45)	MCC950 directly suppresses NLRP3 inflammasome activity. In ischemia complicated by diabetes, MCC950 improved cognitive performance and vasoneuronal remodeling.	Acute/early subacute inflammatory phase; optimal duration remains undefined.	May limit inflammatory amplification and improve selected delayed recovery-related outcomes.	Direct NLRP3 pharmacology with stroke-related preclinical outcome evidence. Recovery evidence is derived from a metabolically comorbid model; clinical efficacy after ischemic stroke remains unproven, and NLRP3 inhibition does not correct upstream lipid burden.
Indirect inflammasome modulation through JAK2/STAT3 (39)	JAK2/STAT3 inhibition reduces NLRP3 activation, neuroinflammation and ischemic injury in experimental stroke.	Primarily acute/early subacute inflammatory phase.	May reduce secondary inflammatory injury and improve neurological outcome.	Stroke-specific preclinical but mechanistically indirect evidence. JAK2/STAT3 is pleiotropic, so treatment effects cannot be attributed exclusively to NLRP3 inhibition.
Recovery-oriented rehabilitation and exercise (43, 46)	Exercise promotes TREM2-dependent microglial support of white matter repair; separate experimental evidence links exercise with reduced inflammasome-related pyroptosis.	Subacute and chronic recovery phases.	Directly relevant to white matter repair, motor recovery and rehabilitation.	Direct stroke-specific experimental recovery evidence. The TREM2 and inflammasome findings arise from separate studies and do not demonstrate an exercise→cholesterol→NLRP3 causal pathway.
Brain- and lesion-targeted delivery platforms (47, 48)	Biomimetic nanomedicine and brain-targeted lipid nanoparticles can enhance delivery of anti-inflammatory agents in experimental stroke.	Potentially applicable across stages depending on therapeutic cargo and target.	May improve brain penetration and cell/lesion targeting of future lipid- or inflammasome-directed therapies.	Stroke-specific preclinical enabling technology. These platforms have not been validated specifically for correcting microglial cholesterol dysregulation or selectively targeting cholesterol-associated NLRP3 signaling.
Stage-specific combination strategies (10, 14, 39–43, 46)	A proposed sequence would restrain excessive early inflammasome-associated inflammation, improve lipid-processing capacity during remodeling, and integrate rehabilitation during recovery.	Acute → subacute → chronic/recovery continuum.	Designed to preserve repair-supportive myeloid functions while addressing temporally distinct inflammatory and metabolic abnormalities.	Therapeutic hypothesis, not a validated regimen. No study identified in this review has directly compared or combined cholesterol-directed therapy, selective NLRP3 inhibition and rehabilitation across predefined post-stroke windows.

**Figure 3:**
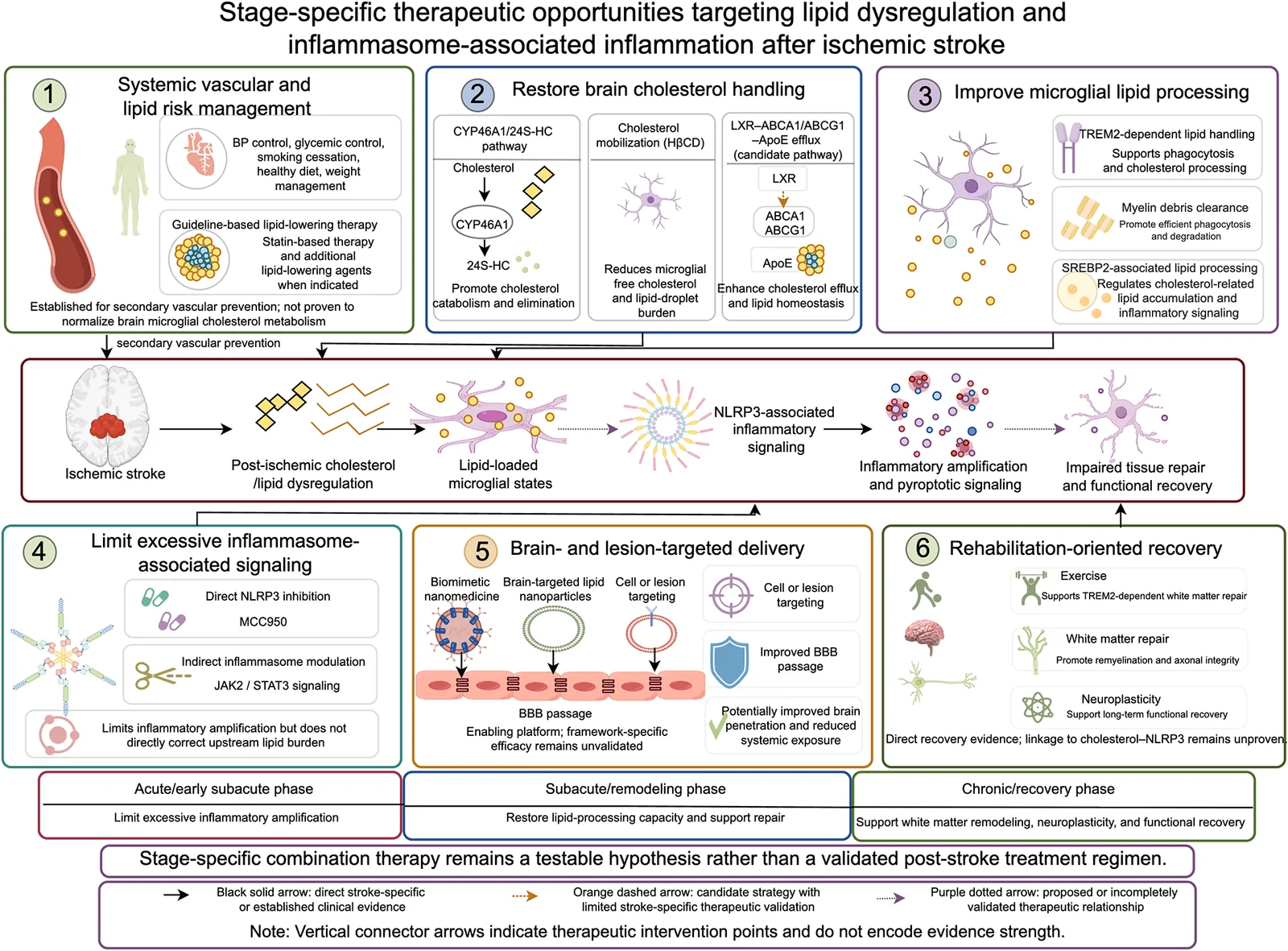
Stage-specific therapeutic opportunities targeting lipid dysregulation and inflammasome-associated inflammation after ischemic stroke. Therapeutic strategies relevant to the proposed cholesterol–microglia–NLRP3 framework differ substantially in target, intervention stage, and level of validation. Systemic vascular and lipid risk management is established for secondary stroke prevention but has not been shown to normalize cholesterol handling within post-ischemic microglia. Among experimental brain-directed approaches, CYP46A1-mediated cholesterol turnover and pharmacological cholesterol mobilization with 2-hydroxypropyl-β-cyclodextrin (HβCD) provide comparatively direct stroke-specific proof-of-concept for reducing cholesterol burden, whereas TREM2- and SREBP2-associated lipid-processing pathways represent additional preclinical targets and LXR–ABCA1/ABCG1–ApoE-mediated efflux remains a mechanistically attractive candidate pathway. Direct NLRP3 inhibition with MCC950 and indirect modulation through JAK2/STAT3 signaling may limit excessive inflammasome-associated inflammatory amplification but do not directly correct upstream lipid-processing abnormalities. Brain- and lesion-targeted delivery systems represent enabling technologies whose specific efficacy for correcting microglial cholesterol dysregulation or cholesterol-associated inflammasome signaling remains unvalidated. Exercise and rehabilitation may support white matter repair, neuroplasticity, and functional recovery, although their mechanistic linkage to the cholesterol–NLRP3 framework remains incompletely established. The proposed timing of interventions across acute/early subacute, subacute/remodeling, and chronic/recovery stages should therefore be regarded as a testable therapeutic framework rather than a validated sequential treatment regimen. Black solid arrows indicate direct stroke-specific or established clinical evidence; orange dashed arrows indicate candidate strategies with limited stroke-specific therapeutic validation; and purple dotted arrows indicate proposed or incompletely validated therapeutic relationships. Vertical connector arrows indicate therapeutic intervention points and do not encode evidence strength. HβCD, 2-hydroxypropyl-β-cyclodextrin; CYP46A1, cholesterol 24-hydroxylase; 24S-HC, 24S-hydroxycholesterol; LXR, liver X receptor; ABCA1/ABCG1, ATP-binding cassette transporters A1/G1; ApoE, apolipoprotein E; TREM2, triggering receptor expressed on myeloid cells 2; SREBP2, sterol regulatory element-binding protein 2; JAK2, Janus kinase 2; STAT3, signal transducer and activator of transcription 3; BBB, blood–brain barrier. By Figdraw.

### 6.5 Translational priorities and unresolved therapeutic questions

Translation of cholesterol- and inflammasome-directed strategies will require stronger evidence across several dimensions. First, the experimental models supporting the proposed framework do not fully reproduce the clinical heterogeneity of ischemic stroke. Much of the direct mechanistic evidence for microglial cholesterol accumulation, lipid droplet remodeling, cholesterol clearance, and inflammasome-associated injury derives from controlled rodent or cellular models (10, 11, 14, 22, 24, 39). Selected studies have incorporated metabolically relevant comorbidities, including diabetes (42), but the effects of aging, dyslipidemia, hypertension, atherosclerosis, sex, prior medication exposure, and multimorbidity on microglial lipid handling and inflammasome responsiveness remain insufficiently characterized. Future preclinical studies should therefore test candidate interventions in aged and comorbidity-relevant models and determine whether therapeutic effects are preserved across biologically distinct stroke contexts.

Second, human validation remains a major limitation. Available clinical studies demonstrate associations between systemic cholesterol-handling measures and short-term stroke outcome and identify compartment-specific lipid signatures in thrombi and plasma (18, 19), but they do not directly measure cholesterol-loaded microglia, cell-specific NLRP3 activity, or the relationship between these processes and long-term neurological recovery. Translational studies should therefore move beyond isolated circulating lipid or inflammatory measurements and integrate complementary biomarkers with longitudinal imaging and functional assessment. Where technically feasible, cerebrospinal fluid or extracellular-vesicle approaches may provide additional information closer to central nervous system processes, although their ability to identify microglia-specific cholesterol–inflammasome states will require direct validation.

Third, therapeutic timing and cellular specificity need to be established experimentally. The pathways considered in this review are unlikely to have identical functions throughout the course of stroke. Early excessive inflammasome-associated signaling may favor transient anti-inflammatory intervention, whereas later recovery increasingly depends on debris clearance, cholesterol processing, white matter remodeling, and repair-supportive microglial functions (10, 40, 41, 43). Furthermore, single-cell and spatial studies demonstrate substantial heterogeneity among post-stroke glial and myeloid states (25–27). Therapeutic strategies should therefore avoid assuming that all microglia should be uniformly suppressed or activated. Instead, future studies should determine which lipid-loaded or inflammasome-prone states are maladaptive, which states support repair, and whether these populations can be targeted without disrupting beneficial immune functions.

Drug delivery represents an additional translational challenge. Brain penetration, regional lesion heterogeneity, and cell-type specificity may limit the effectiveness of both lipid-modifying and inflammasome-targeted agents. Experimental stroke studies using biomimetic nanomedicine or brain-targeted lipid nanoparticles demonstrate that local enhancement of anti-inflammatory drug delivery is technically feasible (47, 48). However, these platforms have not been validated specifically for correcting microglial cholesterol dysregulation or selectively inhibiting cholesterol-associated inflammasome signaling. Delivery technology should therefore be considered an enabling platform rather than evidence that the proposed cholesterol–microglia–NLRP3 framework is already therapeutically targetable in patients.

Finally, therapeutic evaluation should prioritize durable neurological recovery rather than acute molecular or histological improvement alone. Infarct volume, inflammatory markers, and early neurological scores remain useful, but interventions intended to modify microglial lipid handling or inflammasome activity should also be evaluated using motor and cognitive outcomes, white matter integrity, remyelination, neurovascular remodeling, and other measures of long-term repair (10, 14, 40–43). The most informative future studies will combine defined treatment windows, cell-resolved mechanistic measurements, lipid and inflammatory biomarkers, and longitudinal functional outcomes. Such studies are needed to determine whether upstream cholesterol correction and downstream inflammasome modulation are complementary therapeutic approaches and, importantly, whether either provides clinically meaningful benefit beyond established vascular prevention and rehabilitation.

## 7 Conclusions and future perspectives

Ischemic stroke produces substantial remodeling of the brain lipid environment, and accumulating stroke-specific evidence indicates that this process extends beyond a passive consequence of tissue injury. Experimental studies have demonstrated dynamic lipid remodeling, lipid droplet accumulation, persistent microglial cholesterol overload, and the emergence of cholesterol-loaded microglial states after cerebral ischemia (10, 11, 14). Importantly, interventions that enhance cholesterol clearance or mobilization can reduce lipid burden and inflammatory responses and improve white matter integrity and neurological recovery (10, 14). These findings support post-stroke cholesterol dysregulation as a biologically relevant modifier of microglial function and recovery, particularly during the subacute and chronic phases.

The cholesterol–microglia–NLRP3 relationship is nevertheless best regarded as a proposed, evidence-stratified mechanistic framework rather than a validated linear causal axis. Stroke-specific studies support microglial cholesterol accumulation and NLRP3-associated inflammatory changes, but they do not yet establish NLRP3 as the obligatory mediator linking cholesterol overload to tissue injury or recovery failure. Several detailed mechanisms proposed to connect lipid stress with inflammasome activation remain supported partly by macrophage, atherosclerosis, or other neuroinflammatory models, while recent stroke-specific cholesterol-mobilization experiments have identified STING as a more directly validated downstream pathway (13, 14). Moreover, post-stroke microglia are functionally heterogeneous, and distinct microglial populations can promote debris clearance, white matter repair, and neurological recovery (40, 41, 43). Therapeutic interpretation should therefore focus on maladaptive lipid-loaded and inflammasome-prone states rather than on indiscriminate suppression of microglial activity.

Future studies should determine when, where, and in which cell populations cholesterol dysregulation meaningfully contributes to inflammasome activity after stroke. Longitudinal experiments combining cell-resolved lipid measurements with direct assays of inflammasome assembly and activation are needed to establish causal relationships across acute, subacute, and chronic stages. Greater use of aged, metabolically comorbid, and sex-stratified models will be important for improving clinical relevance.

In parallel, human studies should integrate lipid and inflammatory biomarkers with imaging markers of tissue repair and long-term motor and cognitive outcomes, because currently available clinical evidence remains largely associative and does not directly demonstrate a brain microglial cholesterol–NLRP3 mechanism (18, 19). From a therapeutic perspective, future studies should evaluate stage-specific approaches that distinguish established systemic vascular prevention from experimental strategies aimed at correcting brain lipid handling, selectively limiting excessive inflammasome-associated inflammation, and supporting rehabilitation and durable neurological recovery.
